# DLGAP5 Promotes Acute Liver Injury via Hepatocyte Pyroptosis-Driven Macrophage Metabolic Reprogramming and M1 Polarization

**DOI:** 10.7150/ijbs.118024

**Published:** 2025-08-30

**Authors:** Xianzhi Liu, Zhiyuan Chen, Jun Lin, Yifan Lian, Wenxuan Gan, Huajie Liu, Xingxiang Huang, Jiaxin Mei, Tianrong Ma, Zhi Lu, Wei Zeng, Yihang Gong, Shuai Chen, Weiling He

**Affiliations:** 1 Department of Gastroenterology, Xiang'an Hospital of Xiamen University, School of Medicine, Xiamen University, Xiamen, Fujian, 361000, China.; 2 Department of Gastrointestinal Surgery, Xiang'an Hospital of Xiamen University, School of Medicine, Xiamen University, Xiamen, Fujian, 361000, China.; 3 Department of Pediatrics, Xiang'an Hospital of Xiamen University, School of Medicine, Xiamen University, Xiamen, Fujian, 361000, China.; 4 Department of Gastroenterology, Zhongshan Hospital of Xiamen University, Xiamen, Fujian, 361000, China.; 5 Department of Gastroenterology and Hepatology, Tongji Hospital, School of Medicine, Tongji University, Shanghai, 200092, China.; 6 Department of Automation, Tsinghua University, Beijing 100084, China.; 7 Institute for Brain and Cognitive Sciences, Tsinghua University, Beijing, 100084, China.; 8 Department of Hepatic Surgery and Liver Transplantation Center, the Third Affiliated Hospital of Sun Yat-sen University, Organ Transplantation Institute, Sun Yat-sen University, Organ Transplantation Research Center of Guangdong Province, Guangdong Province Engineering Laboratory for Transplantation Medicine, Guangzhou, 510630, China.; 9 Biotherapy Centre & Cell-gene Therapy Translational Medicine Research Centre, the Third Affiliated Hospital, Sun Yat-sen University, Guangzhou, 510630, China.; 10 Department of Digestive Disease, Institute for Microbial Ecology, School of Medicine, Xiamen University, Xiamen, Fujian, 361000, China.

**Keywords:** Acute liver injury (ALI), Hepatocyte (HC), Pyroptosis, DLGAP5, Macrophage, Intercellular communication

## Abstract

Pyroptosis is a novel programmed cell death that exists in inflammatory diseases and methyltransferase-like 3 (METTL3) is a core N6-methyladenosine (m6A) modified methyltransferase that has been shown to regulate cell fate. However, the role of pyroptosis in acute liver injury (ALI) is still unknown and whether it is regulated by m6A modification needs to be elucidated. Here, *Mettl3* mutant and *Nlrp3* knockout mouse were constructed, CCl_4_- and TAA-induced ALI models were established and primary cells were isolated, and cell pyroptosis and m6A modification were evaluated. We found that hepatocyte pyroptosis is a key characteristic of ALI, and METTL3-mediated m6A modification was upregulated in hepatocytes during ALI. Inhibition of METTL3-mediated m6A modification alleviated hepatocyte pyroptosis and ALI. Through MeRIP-seq analysis and verification, *Dlgap5* was determined as the target of METTL3-mediated m6A modification, which was regulated in an IGF2BP2-dependent manner. Mechanistically, METTL3 can bind to DLGAP5, and then DLGAP5 promoted pyroptosis through NF-κB-dependent NLRP3 inflammasome activation and direct potentiation of inflammasome structure formation and assembly. *Mettl3* mutation or AT9283-mediated DLGAP5 inhibition alleviated pyroptosis and ALI. The effects of hepatocyte pyroptosis on cell interaction were then explored and we revealed that NLRP3 inflammasome and interleukin releasing by the GSDMD-N-dependent membrane pores from pyroptotic hepatocytes activated macrophage metabolic reprogramming and M1 polarization, further exacerbating ALI. *Nlrp3* deficiency alleviated ALI by suppressing hepatocyte pyroptosis and blocking communication between macrophages and hepatocytes. Our findings indicate the potential mechanisms of ALI from an intercellular communication perspective, and targeted-inhibition of DLGAP5 and -blockade of hepatocyte-macrophage interaction provide promising strategies for ALI treatment.

## Introduction

Acute liver injury (ALI) is characterized by hepatocyte death and the rapidly expanding inflammatory response 1. It is of great significance to reveal the factors that promote hepatocyte death to prevent the expansion of the injured zone and inhibit inflammation. Pyroptosis is a new type of programmed cell death, characterized by the rupture of the plasma membrane and the release of pro-inflammatory contents 2, occurring in the parenchymal cells such as cardiomyocytes, renal tubular epithelial cells and neurons 3-5. However, the role of pyroptosis in hepatocytes during ALI is still poorly understood.

N6-methyladenosine (m6A) is an important internal post-transcriptional RNA modification in eukaryotic cells 6, regulating RNA splicing, degradation, export and translation, and subsequent protein synthesis 7. m6A modification plays a critical role in embryonic development and immune homeostasis 8-10. Methyltransferase-like 3 (METTL3) is a typical m6A methyltransferase 11. As reported, METTL3-mediated m6A modification respectively promoted acute kidney injury, acute lung injury and acute brain injury via promoting TAB3 expression in an IGF2BP2 dependent manner 12, activating ferroptosis by upregulating ACSL4 under histone acetylation 13, driving neuroinflammation and neurotoxicity through stabilizing *Batf* mRNA in microglia 14, indicating that targeted-METTL3 inhibition was a promising therapy in acute organ injury. However, the effects of METTL3-related m6A modification in ALI have not been fully elucidated, and whether and how hepatocyte pyroptosis participates in the process remains unclear.

Macrophages maintain the tissue homeostasis and about 80% of macrophages are present in the liver 15. Once injured, parenchymal cell releases a series of damage associated molecular patterns (DAMPs) to activate macrophages 16. Activated-macrophage not only directly initiates the immune inflammatory responses by releasing pro-inflammatory cytokines, but also recruits peripheral immune effector cells to migrate to the liver 17. Therefore, regulation of macrophage activity may effectively reduce the degree of liver damage. For example, Du et al. 18 found that hepatic HSPA12A inhibited macrophage chemotaxis and inflammatory activation by suppressing glycolysis mediated-HMGB1 emulsification and hepatic secretion, thereby protecting the liver from ischemia-reperfusion injury. And macrophages can also be activated by hepatocyte mitochondrial DNA to disrupt immune and metabolism homeostasis in the liver 19. Currently, it is not fully understood whether parenchymal cell pyroptosis can activate macrophages in ALI.

In this study, we found that hepatocyte pyroptosis plays an undeniable role in ALI. Mechanistically, METTL3 induces m6A methylation of *Dlgap5* in an IGF2BP2-dependent manner in hepatocytes, and DLGAP5 promotes hepatocyte pyroptosis through NF-κB-dependent NLRP3 inflammasome activation and direct potentiation of inflammasome structural formation and assembly. The pyroptotic hepatocytes not only initiate ALI directly, but also activate macrophage metabolic reprogramming and M1 polarization to exacerbate ALI. Based on these findings, targeted-inhibition of DLGAP5 and -blockade of hepatocyte-macrophage interaction are effective and promising treatment options for ALI.

## Materials and Methods

### Animal experiments

*Mettl3* wild-type (*Mettl3*-WT) and *Mettl3* mutant (*Mettl3*-Mut) mice and *Nlrp3* wild-type (*Nlrp3*-WT) and *Nlrp3* knockout (*Nlrp3*-KO) mice on a C57BL/6 background were established. To construct the *Mettl3*-Mut mice, CRISPR/Cas9-mediated gene editing was employed. In brief, exons 2-3 of the *Mettl3*-201 transcript (ENSMUST00000022767.15) was selected as the knockout target. First, sgRNA was transcribed *in vitro* and co-microinjected Cas9 protein with the sgRNA into fertilized zygotes (one-cell embryos) from C57BL/6 mice. Then, these embryos were transplanted into recipient females to generate F0 offspring. F0-positive mice were screened using polymerase chain reaction (PCR) and DNA gel electrophoresis to confirm *Mettl3* mutation. Finally, F0-positive generation mice were crossed with wild-type C57BL/6 mice to establish a genetically stable F1-generation *Mettl*3 mutant line for our experiments. All mice were male and housed in SPF grade animal house, with free access to water and food. The rearing environment had a light/dark cycle of 12 hours and was maintained at 24±1°C and (50±10) % relative humidity. All mice received adaptive feeding for 7 days before the experiment, and were 6-8 weeks old (18-20g) during the formal experiment. All animal experimental procedures have been approved by the Ethics Committee of Xiamen University and conducted in accordance with the Guide for the Care and Use of Laboratory Animals. For the Carbon tetrachloride (CCl_4_)-induced ALI model, mice were randomly divided into a control group and a CCl_4_ group. The CCl_4_ group was induced by intraperitoneal injection of 1% CCl_4_ (dissolved in an olive oil solution) at 10 mL/kg body weight for 24 hours, while the control group was induced by intraperitoneal injection of an equal amount of olive oil solution. For the Thioacetamide (TAA)-induced ALI model, mice were intraperitoneally injected with 300 mg/kg TAA dissolved in phosphate buffered saline (PBS). The same volume of PBS was intraperitoneally injected into the control mice. For DLGAP5 inhibition, mice received an intraperitoneal injection of AT9283 (20 mg/kg), and the control group was received equal volume of saline intraperitoneal administration.

### Isolation of primary hepatocytes, macrophages, T cells, B cells, NK cells and hepatic stellate cells

Mouse was fixed on the operating table after anesthesia and the indwelling needle was inserted into the inferior vena cava. 10mL of infusion solution A was injected into the mice at a rate of 4 mL/min, and then infusion solution B containing 0.5mg/mL collagenase IV was replaced. During the infusion process, a vascular clamp was applied to temporarily control blood flow. Digestion was terminated when a crack appeared in the liver and gentle pressure did not rebound. The liver was then cut off and immersed into 15mL of fresh medium. The cell suspension was obtained after the liver capsule was torn open to collect naturally flowing cells. For hepatocytes, the cell suspension was centrifuged at 50g for 3 minutes and the precipitate was resuspended and plated in Petri dish. Non-parenchymal cells (NPCs) from the 50g supernatant were collected by centrifugation at 500g for 5 minutes. For macrophages, the NPCs was resuspended in 25% percoll, layered over 50% percoll, and centrifuged at 1600g for 25 minutes. The cells at the interface were collected and plated in Petri dish, and the medium was replaced after 30 minutes. For hepatic stellate cells (HSCs), the NPCs were separated via 8.2% Nycodenz density gradient centrifugation at 1400g for 20 minutes, with low-density auto-fluorescent cells collected. For lymphocytes, the NPCs were purified using Lympholyte®-M Cell Separation Media (Cedarlane), followed by magnetic sorting according to the manufacturers' protocols: B cells: CD19⁺ MicroBeads; NK cells: CD49b⁺ MicroBeads; T cells: CD3^+^ cells (negative selection).

### Cell culture, inhibitor or cytokine treatment

Primary cells were cultured in RPMI medium 1640 (Gibco) supplemented with 10% fetal bovine serum (FBS). Cells were cultured in a humidified incubator at 37°C and 5% CO_2_. For co-culture system, primary macrophages were isolated from the livers of mice and seeded at transwell insert (Corning) for overnight culture. After adherence, the insert was placed on the top of primary hepatocytes isolated from mice with different treatments, and co-cultured for 24 hours. Primary macrophage lysate was harvested for further experiments, and cells were also fixed with 4% paraformaldehyde for staining. To explore the role of NF-κB pathway in the activation of NLRP3 inflammasome, primary hepatocytes were treated with 10 μM NF-κB pathway inhibitor Bay117082. To study the role of IL-1β and IL-18 in macrophage M1 polarization, recombinant IL-1β protein (10 ng/mL) and recombinant IL-18 protein (20 ng/mL) were added into the co-culture system containing primary macrophages and primary hepatocytes.

### Transfection of plasmids and siRNA

For *Dlgap5* overexpression, the full-length *Dlgap5* coding sequence was cloned into pcDNA3.1 vector for transient transfection using Lipofectamine 3000. For domain interaction analysis, truncated constructs encoding defined structural domains including NLRP3-PYD (UniProt: Q8R4B8, residues 1-91), ASC-PYD (UniProt: Q9EPB4, residues 1-91), ASC-CARD (residues 105-193), and CASPASE1-CARD (UniProt: P29452, residues 1-91) were subcloned into pcDNA3.1 vectors. The constructs were transfected into hepatocytes for further experiment. For targeted gene silencing, the small interfering RNA (siRNA) specifically targeting *Caspase1* (si-*Casp1*), *Mettl3 (*si-*Mettl3)*, *Dlgap5 (*si-*Dlgap5)*, *Nlrp3* (si-*Nlrp3)*, *Il-18 (*si-*Il-18)*,* Il-1β (*si-*Il-1β)* and the corresponding non-targeting control siRNA (si-*Con*) were separately introduced using the Lipofectamine 3000 according to the manufacturer's instruction.

### Serum transaminase activity

Mouse blood samples were centrifuged at 4000 rpm for 10 minutes at 4°C, and the supernatant was collected and stored at -80°C. The activities of alanine aminotransferase (ALT) and aspartate aminotransferase (AST) in serum were measured using indicated kits according to the manufacturer's instructions.

### PI staining

After treatments, primary hepatocytes were isolated and washed with Phosphate Buffered Saline (PBS) and then incubated with PI staining solution at 4°C in the dark for 20-30 minutes, followed by counterstaining with corresponding antibody to achieve PI/Cleaved CASPASE1 co-staining. Then Cells were observed under a fluorescence microscope.

### H&E, immunohistochemistry (IHC), immunofluorescence (IF) and TUNEL staining

Fresh mouse liver tissues were fixed in 4% paraformaldehyde solution. Fixed liver tissue was dehydrated, embedded in paraffin, and sliced into 4-5 μm sections for preservation. Before the experiment, the sections were taken out and subjected to deparaffinized and hydrated treatment. For H&E staining, the sections were stained with hematoxylin-eosin, dehydrated, dried, sealed with neutral gum, and then observed under a microscope. For IHC staining, sections were subjected to antigen repair in boiling sodium citrate repair solution. Endogenous peroxidase inhibitors were added dropwise and soaked the tissue for 10 minutes. The corresponding primary antibodies were added and incubated overnight at 4°C. On the second day, the secondary antibody was added and incubated at 37°C for 2 hours, followed by color development using freshly prepared diaminobenzidine (DAB) color solution according to the instructions. For IF staining, the sections were incubated with the fluorescent secondary antibodies and stained with DAPI to mark the nuclei. The staining was observed under a fluorescence microscope. For TUNEL staining, sections were incubated with TUNEL staining solution in the dark for 60 minutes, then placed in the DAPI solution in the dark for 10 minutes, followed by counterstaining with corresponding antibody to achieve TUNEL/GSDMD-N co-staining. After adding anti-fade mounting medium and covering with a cover glass, sections were observed under a fluorescence microscope.

### Western blot analysis

Total protein was extracted from the tissues or cells using RIPA lysis buffer containing protease inhibitors, and protein concentration was measured using BCA protein quantification kit. After boiled and denatured, equal amounts of the protein sample were separated by 10% SDS-PAM gel, transferred to polyvinylidene fluoride (PVDF) membrane, blocked with skim milk, and then incubated overnight at 4 °C with corresponding primary antibodies. The next day, the PVDF membrane was incubated with HRP-conjugated secondary antibodies at room temperature for 1 hour, then developed using electrochemiluminescence method.

### Enzyme‑Linked Immunosorbent Assay (ELISA)

The levels of IL-1β, IL-18 and Caspase1 were determined using corresponding ELISA kit according to the manufacturer's instruction. The absorbance was measured using a microplate reader and the concentration of each sample was calculated based on the standard curve.

### Real-time quantitative Polymerase Chain Reaction (RT-qPCR)

Total RNA was extracted from the tissues or cells using the TRIzol reagent and reverse-transcribed to cDNA. RT-qPCR was then performed using specific primers and SYBR Green mix (Vazyme). The specificity of the products was verified according to the dissolution curve analysis, and the relative expression of the genes was calculated according to the 2^-ΔΔCt^ method.

### m6A dot-blot assay

RNA samples were mixed with prepared denaturing solution by equal volume and denatured at 95°C for 5 mins, followed by spotting onto a nitrocellulose membrane. Samples were then UV crosslinked under a 302 nm ultraviolet lamp. The cross-linked samples were blocked at room temperature with 5% skim milk for 2 hours, and then incubated overnight with m6A antibody at 4°C. The next day, corresponding secondary antibody was added to the blots and incubated at room temperature for 2 hours, then developed using electrochemiluminescence method.

### RNA immunoprecipitation (RIP) assay

RIP assay was performed using the RNA-binding protein immunoprecipitation kit. Briefly, magnetic beads were incubated with IGF2BP2 antibody or IgG antibody for 30 min. The pretreated magnetic beads were mixed with prepared cell lysates and incubated at 4°C overnight. Next day, the RNA-protein complexes were washed and proteinase K digestion buffer was added to remove the proteins. RNA was finally extracted and quantified by RT-qPCR.

### Co-immunoprecipitation (Co-IP) assay

Cells were lysed in Pierce™ IP Lysis Buffer and precleared with protein A magnetic beads (Bio-ray) overnight at 4°C, then incubated with indicated antibody for 2 h at room temperature. Finally, magnetic beads were washed and boiled for 10 min at 70°C and harvested for WB analysis. Anti-IgG antibody was used as a control.

### Determination of oxidative stress

For DHE staining, liver tissue sections were incubated with DHE staining solution in the dark for 30 minutes. After adding anti-fade mounting medium and covering with a cover glass, sections were observed under a fluorescence microscope. For the detection of MDA, SOD, GSH and GSSG levels, after extracting liver tissue homogenate, corresponding reagent kit were used according to the manufacturer's instructions. The ROS levels were measured with a kit (Beyotime Biotechnology), and 2,7-Dichlorodihydrofluorescein diacetate was used to incubate the cells and then examined by microplate reader.

### Molecular docking

The protein or chemical 3D structures of DLGAP5, METTL3, NLRP3 and AT9283 were obtained by searching the RCSB PDB (https://www.rcsb.org/) and PubChem databases (https://pubchem.ncbi.nlm.nih.gov). Protein-protein docking was performed using Hdock server (http://hdock.phys.hust.edu.cn/), and protein-chemical docking was performed using AutoDock 4.2 and AutoDock tools 1.5.7. PyMOL (https://www.pymol.org/) was used to visualize the results.

### RNA sequencing (RNA-seq)

Primary macrophages were isolated from indicated mice and total RNA was extracted. Sequencing libraries were prepared according to the manufacturer's instruction with steps including, purifying mRNA and fragmenting into small pieces, synthesizing first- and second- strand cDNA, performing end repair by incubation with A-Tailing Mix, and ligating RNA Index Adapters. The products were purified and dissolved, then validated for quality control. The final library was amplified with phi29 to make DNA nanoballs (DNBs) and then loaded into a patterned nanoarray, and single-end 50-base reads were generated on the MGIseq2000 platform. Raw reads were processed through adapter trimming, HISAT2 alignment (mm10 genome), and gene quantification with DESeq2 for differential expression analysis.

### Glycolysis analysis

Glucose uptake, lactate production and ATP levels were detected by corresponding kits according to the manufacturers' protocols. For seahorse experiment, XFe96 Extracelluar Flux Analyzer was used to measure oxygen consumption rate (OCR) and extracellular acidification rate (ECAR) using the Cell Mito Stress Test Kit (Agilent) and the Glycolysis Stress Test Kit (Agilent) respectively. For OCR, 1.5 μmol/L oligomycin (ATP synthase inhibitor), 2 μmol/L FCCP (mitochondrial uncoupler) and 0.5 μmol/L rotenone/antimycin A (Complex I/III inhibitors) were sequentially added to the culture plate and maximal respiration and non-mitochondrial respiration values derived from OCR kinetics were recorded. For ECAR, following baseline recording, cells were exposed to sequential injections of 10 mM glucose (to induce glycolysis), 1.5 μmol/L oligomycin (to inhibit mitochondrial ATP production and force maximum glycolytic capacity), and 100 mM 2-deoxy-D-glucose (2-DG, a glycolysis inhibitor). Sample Preparation Protocol: Cells were equilibrated in pre-warmed (37°C) medium containing 10 mM glucose, 1 mM pyruvate, and 2 mM glutamine. Prior to assay initiation, cultures underwent a 60-min CO₂-free incubation at 37°C to normalize pH. All reagents were prepared in pH-adjusted medium and sterile-filtered (0.22 μm) before loading into sensor cartridges.

### Public data analysis

The data that support the findings of this study can be found in the GEO data repository (https://www.ncbi.nlm.nih.gov/geo/), including the accession numbers GSE166868, GSE45002, GSE211460, GSE113756, GSE135462 and GSE223558. All statistical analyses were conducted using the R (version 4.4.1) or SAS 9.4 statistical software.

### Statistical analyses

The results were expressed as the means ± standard errors of the means. Student's two-tailed paired *t*-test, one-way analysis of variance (ANOVA) or two-way ANOVA were carried out. *p* < 0.05 was considered to indicate statistical significance.

## Results

### Pyroptosis is involved in acute liver injury

**Figure 1:**
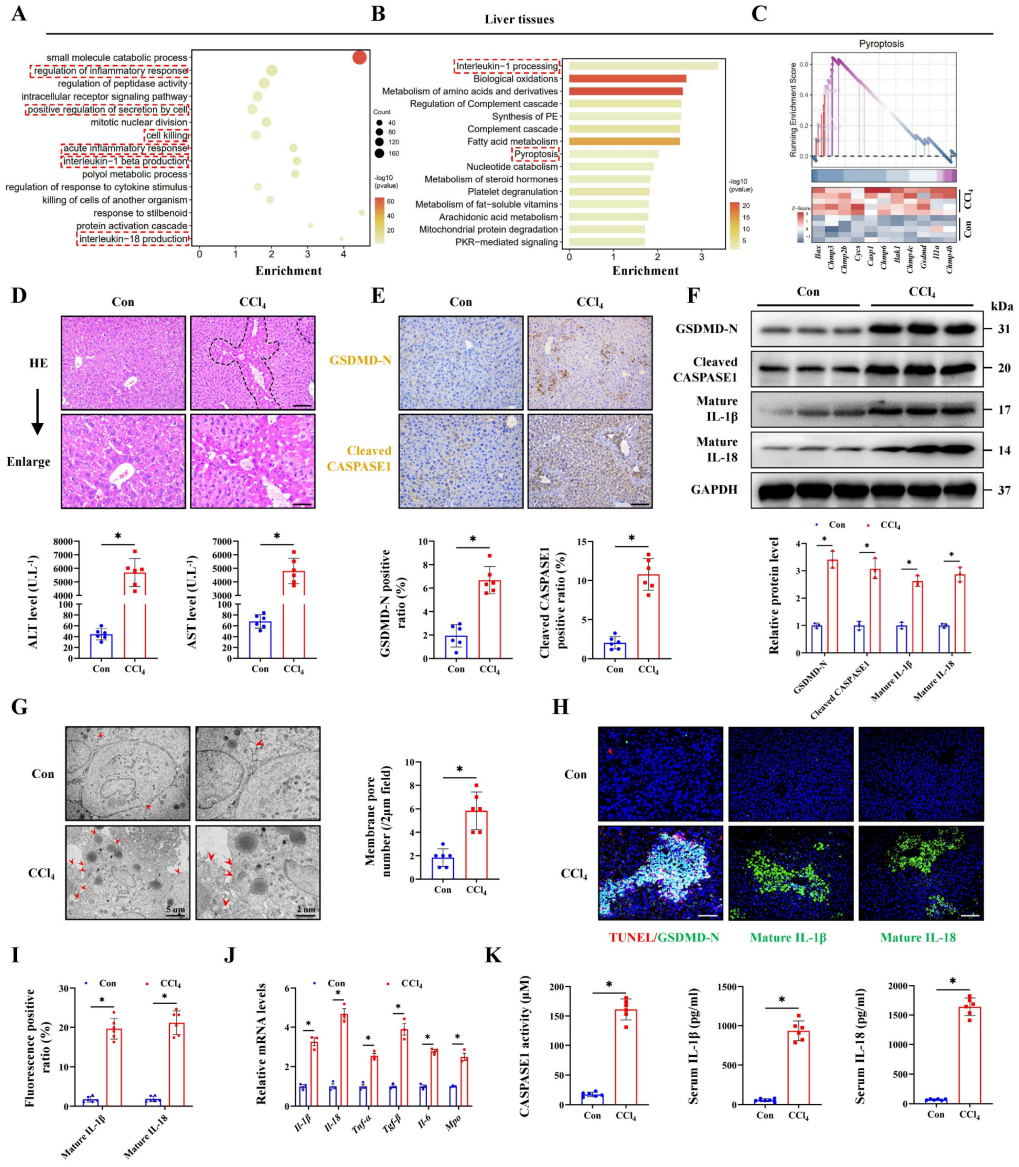
**Pyroptosis is involved in acute liver injury. (A)** Signaling pathways related to inflammation and cell death were screened by the GO enrichment analysis. **(B)** Reactome enrichment analysis based on differentially expressed genes (DEGs). **(C)** GSEA enrichment plot and heatmap showing upregulation of pyroptosis-related signaling in CCl_4_ group compared to control group. **(D)** H&E staining in liver sections (Scale bar: 50μm, 25μm) and serum ALT and AST levels of control group and CCl_4_ group (n = 6 per group). **(E)** IHC staining of GSDMD-N and Cleaved CASPASE1 in liver sections from indicated groups (Scale bar: 50μm, n = 6 per group). **(F)** Protein expression of GSDMD-N, Cleaved CASPASE1, mature IL-1β and mature IL-18 in mouse liver treated with or without CCl_4_ (n = 3 per group). **(G)** Ultrastructural features of liver identified by TEM under control and CCl_4_ treatment (Scale bar: 5μm, 2μm, n = 6 per group).** (H, I)** Co-IF staining of TUNEL/GSDMD-N (Scale bar: 25μm) and IF staining of mature IL-1β and mature IL-18 (Scale bar: 50μm) in liver sections from indicated groups (n = 6 per group). **(J)** mRNA levels of *Il-1β*, *Il-18*, *Tnf-α*, *Tgf-β*, *Il-6* and *Mpo* in liver tissues were determined by RT-qPCR (n = 3 per group). **(K)** The levels of CASPASE1 activity, IL-1β and IL-18 in serum from indicated group were measured (n = 6 per group). All results are shown as mean ± SEM. *p < 0.05.

To explore the genetic characteristics of ALI, RNA transcriptome data from mice treated with CCl_4_ was analyzed. Gene Ontology (GO) analysis indicated that the inflammatory response and IL-1β/IL-18 production were activated in ALI, together with cell killing pathway upregulation (Figure 1A). Reactome enrichment and GSEA analysis further intuitively demonstrated the significant activation of the pyroptosis signaling during ALI (Figure 1B, 1C). Next, experimental results indicated that CCl_4_ treatment promoted necrosis and inflammatory infiltration compared with the control group, and the serum ALT and AST levels were also significantly increased (Figure 1D, 1E). To verify whether pyroptosis was involved in the pathogenesis of ALI, we performed IHC staining and results showed the increased expression of pyroptosis marker GSDMD-N and Cleaved CASPASE1 in the CCl_4_ group. The protein levels of GSDMD-N and Cleaved CASPASE1 were also increased in the CCl_4_ group accordingly (Figure 1F). Cell swelling and the formation of membrane pore observed by transmission electron microscopy (TEM) were also increased after CCl_4_ treatment (Figure 1G). TUNEL and GSDMD-N co-staining was further used to accurately label pyroptosis cells, and the results confirmed that the number of pyroptosis cells in the CCl_4_ group was significantly higher than that in the control group (Figure 1H, 1I). Meanwhile, we verified the expression of downstream molecules of the pyroptosis pathway from multiple dimensions. Compared with the control group, the mRNA expressions of *Il-1β*, *Il-18*, *Tnf-α*, *Tgf-β*, *Il-6* and *Mpo* in the CCl_4_ group were all increased (Figure 1J). Significant increases of serum IL-1β and IL-18 levels and CASPASE1 activity in the CCl_4_ group were confirmed (Figure 1K). Both WB and IF results showed that CCl_4_ treatment elevated the levels of mature IL-1β and IL-18 (Figure 1F, 1H, 1I). To further validate the cell pyroptosis during ALI, we examined in a second thioacetamide (TAA)-induced ALI model, and found the consistent pyroptosis phenomenon (Figure S1A-S1H). These data all above suggest that pyroptosis is involved in the pathogenesis of ALI.

### Hepatocyte pyroptosis is the critical feature of acute liver injury

**Figure 2:**
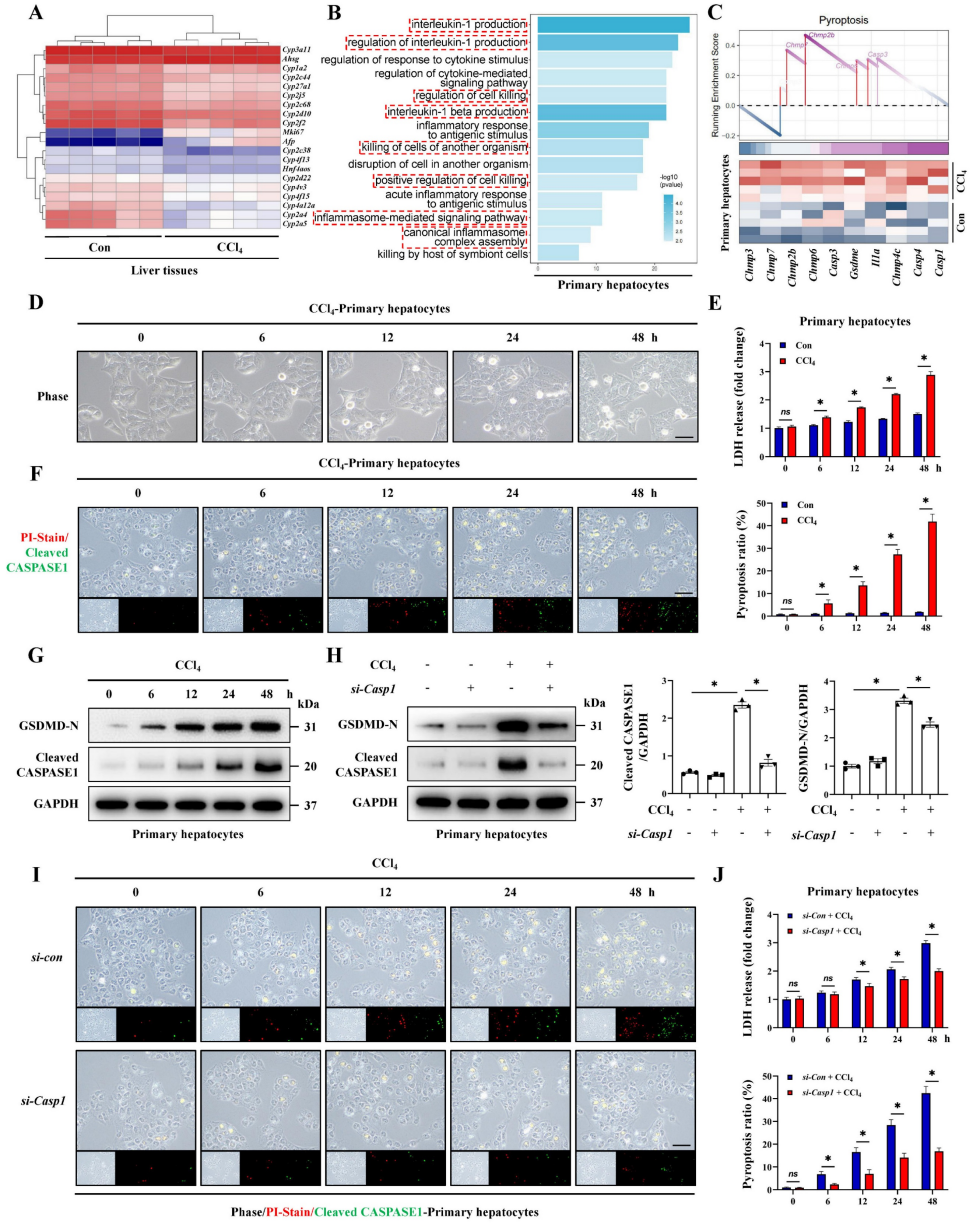
** Hepatocyte pyroptosis is the critical feature of acute liver injury. (A)** Heatmap of DEGs related to hepatocyte injury in whole liver tissue sequencing. **(B)** Signaling pathways related to inflammation and cell death were enriched by GO analysis. **(C)** GSEA enrichment plot and heatmap showing upregulation of pyroptosis-related signaling in the primary hepatocytes from CCl_4_ group compared to the control group. **(D)** Phase contrast micrographs of primary hepatocytes from mice treated with CCl_4_ at 0, 6, 12, 24, and 48 hours (Scale bar: 50μm). **(E)** LDH activity in the cell culture supernatant at different timeline under control and CCl_4_ treatment (n = 3 per group). **(F)** PI/Cleaved CASPASE1 co-staining of primary hepatocytes at different timeline of the CCl_4_ groups (Scale bar: 50μm, n = 3 per group). **(G)** Protein levels of GSDMD-N and Cleaved CASPASE1 in primary hepatocytes from CCl_4_-treated mice at different timeline. **(H)** Protein levels of GSDMD-N and Cleaved CASPASE1 in primary hepatocytes transfected with si-*Caspase1* and control si-RNA from indicated groups (n = 3 per group). **(I, J)** PI/Cleaved CASPASE1 co-staining of primary hepatocytes and LDH activity in the cell culture supernatant at different timeline from indicated groups (Scale bar: 50μm, n = 3 per group). All results are shown as mean ± SEM. *p < 0.05. ns, not significant.

We noticed that a large number of hepatocyte-related genes showed significant differences during ALI (Figure 2A). Therefore, we further explored the characteristics of hepatocytes in ALI. The transcriptome results regarding GO/KEGG enrichment and GSEA analysis of hepatocytes from CCl_4_-induced ALI model suggested that pyroptosis was likely to occur in hepatocytes during ALI (Figure 2B, 2C, S2A). Next, we extracted primary hepatocytes from ALI models and found that the pyroptosis pathway was significantly activated in primary hepatocytes from ALI mice compare to control mice (Figure S2B-S2E). To highlight this, we established dynamic timeline ALI models using CCl_4_ and TAA respectively and primary hepatocytes were isolated at 0, 6, 12, 24 and 48 hours post-administration for culture and observation. We found that the LDH activity in the cell culture supernatant and the ratio of PI/Cleaved CASPASE1 co-positive pyroptotic hepatocytes from both CCl_4_ and TAA model increased continuously (Figure 2D-2F, S1I-S1K). In addition, with the prolongation of above intervention, the levels of GSDMD-N and Cleaved CASPASE1 increased gradually (Figure 2G). Activated CASPASE1 is a key upstream molecule initiating pyroptosis, we thus suppressed *Caspase1* by siRNA and results showed that the previously upregulated-GSDMD-N decreased after *Caspase1* inhibition (Figure 2H). Meanwhile, LDH activity and the pyroptotic hepatocytes also showed a significant decrease by *Caspase1* inhibition (Figure 2I, 2J). The results indicate that CASPASE1/GSDMD mediated- hepatocyte pyroptosis is the critical feature of ALI.

### Hepatocyte pyroptosis is regulated by METTL3-mediated m6A methylation

**Figure 3:**
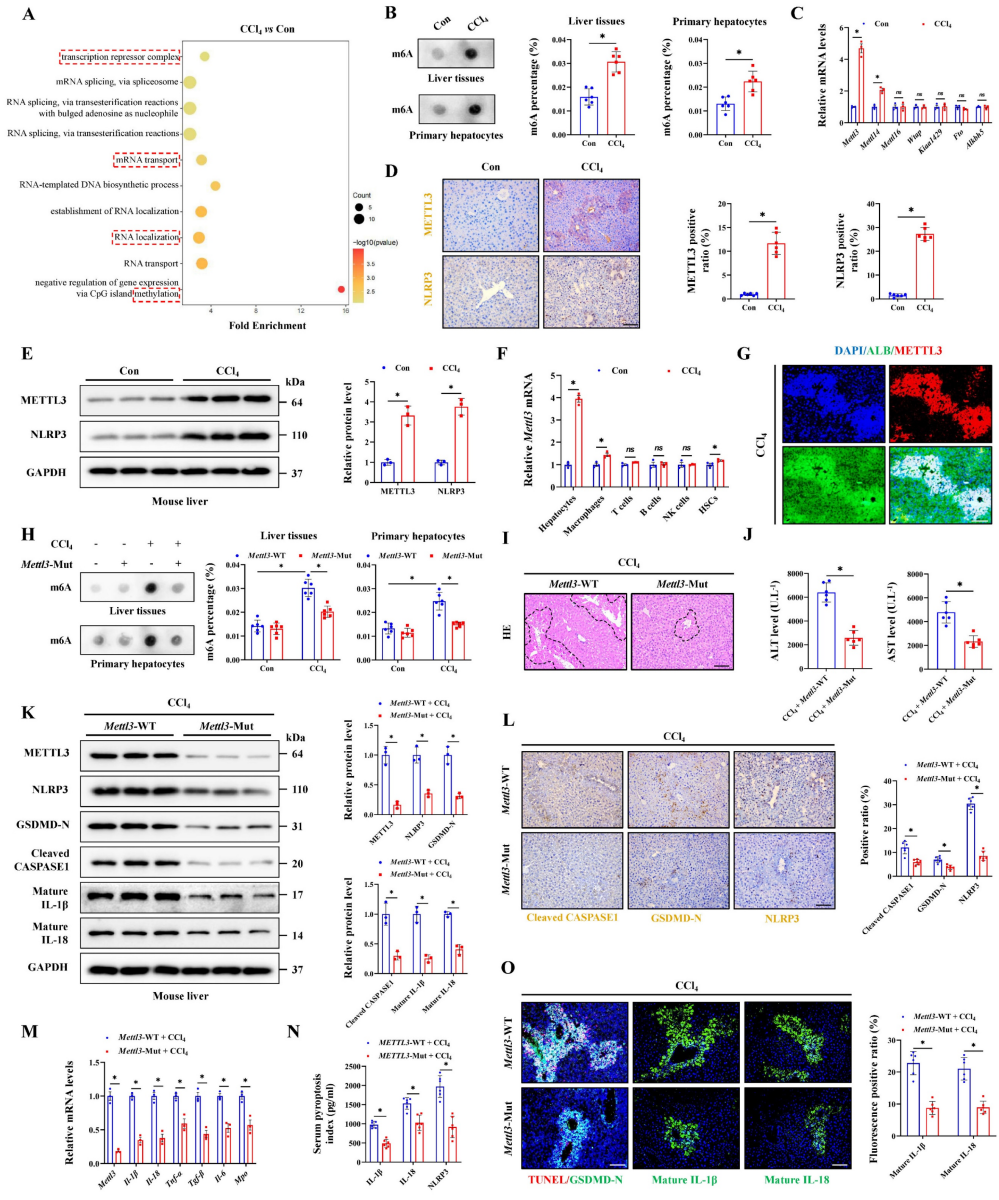
** Hepatocyte pyroptosis is regulated by METTL3-mediated m6A methylation. (A)** Signaling pathways related to RNA modification and methylation were enriched. **(B)** The m6A level of liver tissues and primary hepatocytes was detected (n = 6 per group). **(C)** mRNA levels of *Mettl3*, *Mettl14*, *Mettl16*, *Wtap*, *Kiaa1429*, *Fto* and *Alkbh5* in primary hepatocytes from the control and CCl_4_ groups were determined by RT-qPCR (n = 3 per group).** (D)** IHC staining of METTL3 and NLRP3 under control and CCl_4_ treatment (Scale bar: 50μm, n = 6 per group). **(E)** Protein expression of METTL3 and NLRP3 under control and CCl_4_ treatment (n = 3 per group). **(F)** mRNA levels of *Mettl3* in primary hepatocytes, macrophages, T cells, B cells, NK cells and HSCs from control or CCl_4_-treated mice (n = 3 per group). **(G)** Co-IF of METTL3 and ALB under CCl_4_ treatment (Scale bar: 25μm). **(H)** The m6A levels of liver tissues and primary hepatocytes were decreased by METTL3 mutation (n = 6 per group). **(I)** H&E staining in liver sections from *Mettl*3-WT or *Mettl3*-Mut mice treated with CCl_4_ (Scale bar: 50μm). **(J)** Serum ALT and AST levels from indicated groups (n = 6 per group). **(K)** Western blot showed the decreased levels of METTL3, NLRP3, GSDMD-N, Cleaved CASPASE1, mature IL-1β and mature IL-18 in *Mettl3*-Mut mice compared to *Mettl3*-WT mice (n = 3 per group). **(L)** IHC staining of NLRP3, GSDMD-N, Cleaved CASPASE1 in liver sections from indicated groups (n = 6 per group).** (M)** mRNA levels of *Mettl3*, *Il-1β*, *Il-18*, *Tnf-α*, *Tgf-β*, *Il-6* and *Mpo* in liver tissues from *Mettl*3-WT or *Mettl*3-Mut mice treated with CCl_4_ (n = 3 per group). **(N)** Serum pyroptosis index from indicated groups (n = 6 per group). **(O)** IF staining of mature IL-1β and mature IL-18 (Scale bar: 50μm) and TUNEL/GSDMD-N co-staining (Scale bar: 25μm) in liver sections from indicated groups (n = 6 per group). All results are shown as mean ± SEM. *p < 0.05. ns, not significant.

Meanwhile, RNA transport, localization and methylation were significantly enriched in injured hepatocytes (Figure 3A), suggesting that RNA methylation may play an important role in pyroptosis process. To identify the potential role of m6A modification in ALI, dot-blot analysis found that the m6A levels in liver tissues and primary hepatocytes from CCl_4_ group were significantly higher than those in the control group (Figure 3B). The m6A modification is mainly deposited by “Writer” (METTL3, METTL14, METTL16, WTAP, and KIAA1429) and removed by “Eraser” (ALKBH5 and FTO). The results found that *Mettl3* showing the largest difference in the ALI group compared to the control group (Figure 3C). Further WB and IHC assays showed that the CCl_4_ treatment upregulated the expression of METTL3 (Figure 3D, 3E). NLRP3 inflammasome activation is the core link in initiating the classical pyroptosis pathway and which was indeed up-expression after CCl_4_ treatment (Figure 3D, 3E). We then isolated various parenchymal and nonparenchymal cells (including hepatocytes, HSCs, macrophages, T cells, B cells and NK cells) for subcellular expression analysis of *Mettl3*. The results showed that hepatocytes were primarily responsible for the upregulation of *Mettl3* under CCl_4_ treatment (Figure 3F). The co-staining of METTL3 and hepatocyte-specific marker albumin (ALB) also confirmed that (Figure 3G). Next, *Mettl3*-mutant mouse was further established and verified by DNA gel electrophoresis (Figure S3A). In whether control or CCl_4_-treated mice, WB and RT-qPCR assays confirmed a significant decrease in METTL3 expression from protein and mRNA levels in both liver tissue and primary hepatocytes in *Mettl3*-mutant mice compared to *Mettl3*-WT mice (Figure 3K, 3M, S3B, S3C, S3E, S3F). Basing that, we measured the level of RNA m6A methylation in total liver tissues and primary hepatocytes. As shown, when *Mettl3* was inhibited, CCl_4_ treatment-mediated m6A level was suppressed (Figure 3H). And the degree of ALI in *Mettl3*-mutant mice was also reduced, accompanied by a smaller area of liver necrotic lesion, lower levels of serum ALT and AST, and fewer hepatocyte pyroptosis compared to WT mice (Figure 3I, 3J, 3O). Moreover, the expression of GSDMD-N, Cleaved CASPASE1, matured IL-1β, matured IL-18 and other inflammatory factors in *Mettl3*-mutant mice were decreased compared to WT mice (Figure 3K-3O). At the cellular level, CCl_4_ treatment gradually promoted the expression of METTL3 and NLRP3 over time (Figure S3D). After inhibition of *Mettl3* in primary hepatocytes, indicators related to pyroptosis were also blocked (Figure S3E-S3I), which indicated that hepatocyte pyroptosis is regulated by METTL3-mediated m6A methylation, targeted-inhibition of METTL3 shows potential to reduce hepatocyte pyroptosis for liver injury attenuation.

### METTL3-mediated m6A methylation promotes DLGAP5 expression in an IGF2BP2-dependent manner

**Figure 4:**
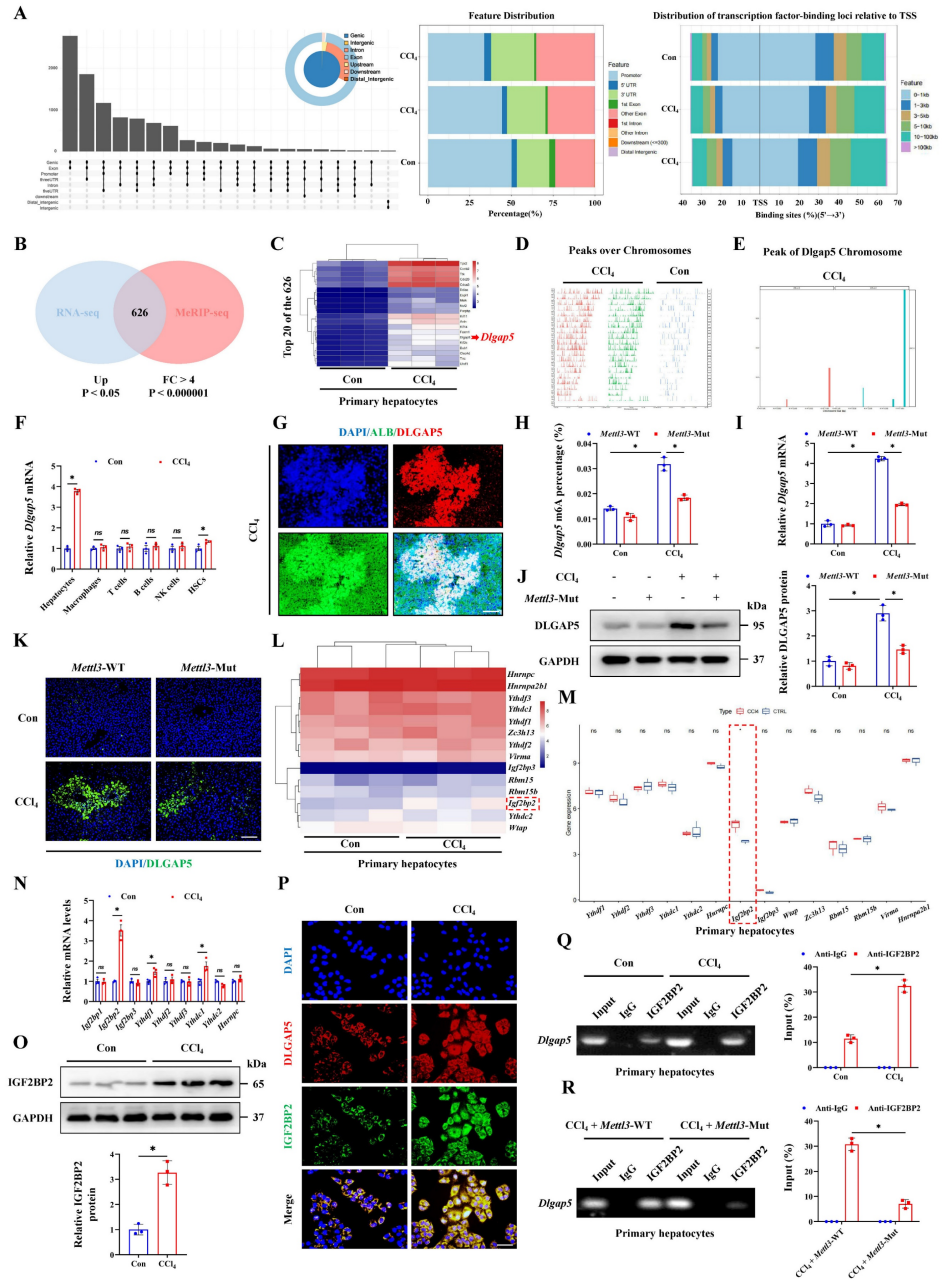
** METTL3-mediated m6A methylation promotes DLGAP5 expression in an IGF2BP2-dependent manner. (A)** The distribution of peaks on gene functional elements according to pie and UpSet plot analyses. **(B)** The Venn diagram showed the overlapping genes generated by the intersection of DEGs extracted from RNA-seq and MeRIP-seq. **(C)** Heatmap of the top 20 of the 626 overlapping genes. **(D)** Distribution of reads on chromosomes in CCl_4_ group relative to those in Control group.** (E)** Peak regions of DLGAP5 over chromosomes. **(F)** mRNA levels of *Dlgap5* in primary hepatocytes, macrophages, T cells, B cells, NK cells and HSCs from control or CCl_4_-treated mice (n = 3 per group). **(G)** Co-IF of DLGAP5/ALB under CCl_4_ treatment (Scale bar: 25μm).** (H)** *Dlgap5* m6A levels in indicated groups (n = 3 per group). **(I)** mRNA level of *Dlgap5* in *Mettl*3-WT and *Mettl*3-Mut mice under control and CCl_4_ treatment (n = 3 per group). **(J)** Protein levels of DLGAP5 in *Mettl*3-WT and *Mettl*3-Mut mice under control and CCl_4_ treatment (n = 3 per group). **(K)** IF staining of DLGAP5 in liver sections from indicated groups (Scale bar: 50μm). **(L, M)** Heatmap and gene expression analysis of potential m6A readers in control and CCl_4_-treated mice according to RNA-seq. **(N)** mRNA levels of m6A readers in primary hepatocytes under control and CCl_4_ treatment (n = 3 per group). **(O)** Protein expression levels of IGF2BP2 (n = 3 per group). **(P)** Co-IF of DLGAP5 and IGF2BP2 in primary hepatocytes from control and CCl_4_ mice (Scale bar: 25μm). **(Q)** RIP assays in primary hepatocytes presented the direct binding between IGF2BP2 and DLGAP5 (n = 3 per group). **(R)** *Mettl3* mutation reduced the binding of IGF2BP2 and DLGAP5 in primary hepatocytes (n = 3 per group). All results are shown as mean ± SEM. *p < 0.05. ns, not significant.

To clarify the specific targets of METTL3 in ALI, we analyzed ‌MeRIP-sequencing data. After CCl_4_ treatment, a great number of genes underwent methylation, and the peak over chromosomes were significantly different from the control group (Figure 4A, 4D). Through taking the intersection of these methylated genes of ‌MeRIP-seq and upregulated genes obtained from RNA-seq of primary hepatocytes, 626 co-regulated genes were identified (Figure 4B), and *Dlgap5* was the gene with the most significant difference (Figure 4C). Under CCl_4_ treatment, *Dlgap5* mRNA underwent methylation (Figure 4E). Thus, *Dlgap5* was regarded as a candidate gene for subsequent validation. We further investigated the cell specific source of the up-regulated *Dlgap5* in ALI by isolating various parenchymal and nonparenchymal cells from the control or ALI mice. The results showed that hepatocytes were the main contributors to the upregulation of *Dlgap5* under ALI (Figure 4F). The co-staining results of DLGAP5 and ALB further confirmed this finding (Figure 4G). Then, we found that the *Dlgap5* m6A level in the CCl_4_ group was significantly increased compared to the control group, while was inhibited by *Mettl3* mutation (Figure 4H), and RT-qPCR, WB and IF assays showed the same trend (Figure 4I-4K). Meanwhile, m6A was regulated by the specific readers, which can alter the stability or translation speed of methylated mRNA 20. We thus analyzed the reader profile in the transcriptome of primary hepatocytes treated with CCl_4_ and noticed that *Igf2bp2* significantly upregulated and was the only one with statistical differences (Figure 4L, 4M). RT-qPCR and WB confirmed the changes of IGF2BP2 during ALI (Figure 4N, 4O). IF staining indicated the co-localization of DLGAP5 and IGF2BP2 expression in primary hepatocytes (Figure 4P). RIP experiments further showed that CCl_4_ treatment promoted the binding of *Dlgap5* and IGF2BP2, while was inhibited by *Mettl3* mutation (Figure 4Q, 4R). Taken together, these results indicate that METTL3-mediated m6A modification targets *Dlgap5* expression in an IGF2BP2-dependent manner, regulating acute hepatocyte and liver injury mechanistically.

### METTL3 promotes hepatocyte pyroptosis in acute liver injury by activating DLGAP5

**Figure 5:**
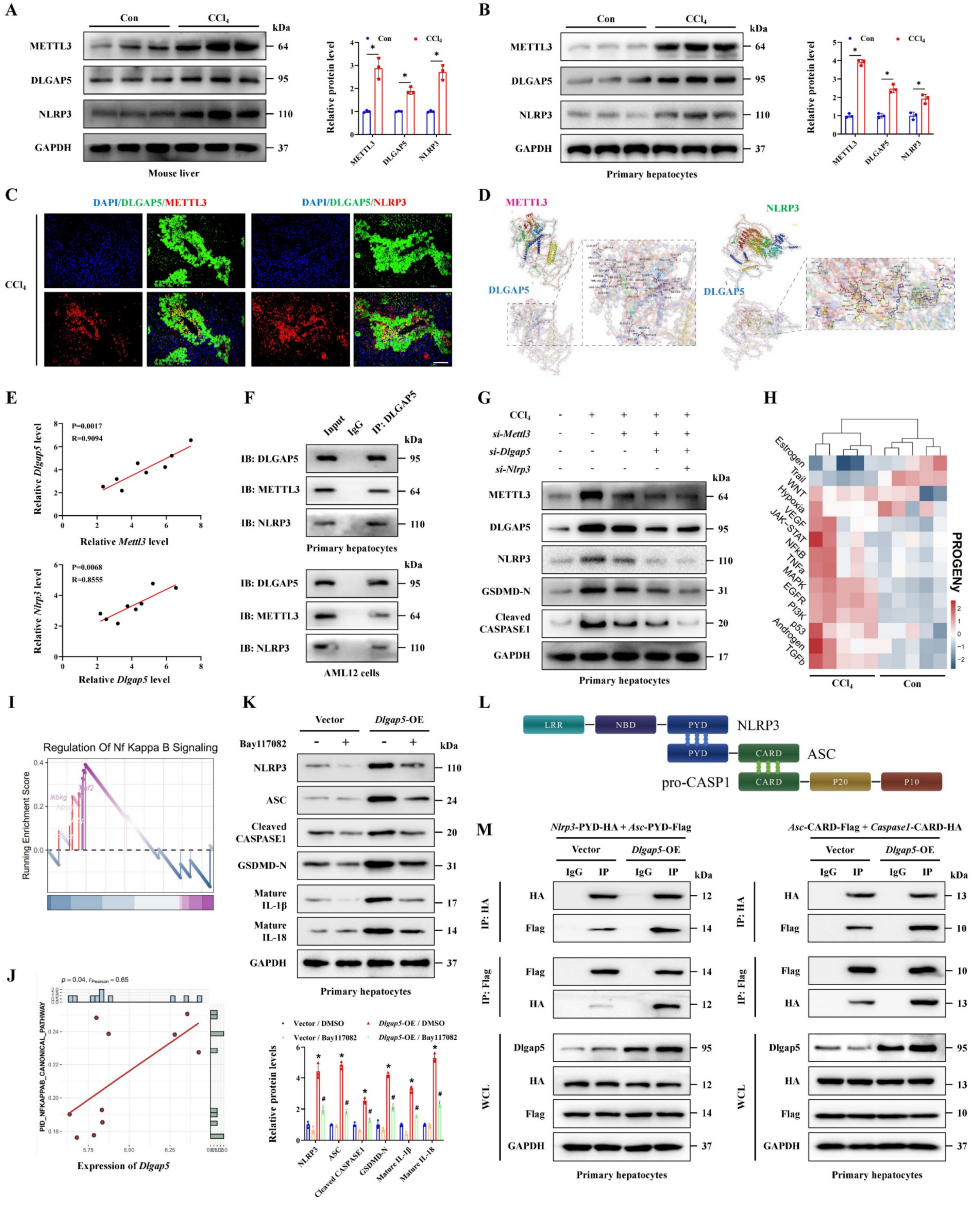
** METTL3 promotes hepatocyte pyroptosis in acute liver injury by regulating DLGAP5. (A, B)** Protein levels of METTL3, DLGAP5 and NLRP3 in liver tissues and primary hepatocytes from mice treated with or without CCl_4_ (n = 3 per group).** (C)** Co-IF staining of METTL3 and DLGAP5, DLGAP5 and NLRP3 in liver sections with CCl_4_ treatment (Scale bar: 50μm). **(D)** Molecular docking of the DLGAP5 protein to the METTL3 and NLRP3 protein. **(E)** Correlation analysis of mRNA levels between *Dlgap5* and *Mettl*3, *Dlgap5* and *Nlrp3* in acute liver injury. **(F)** Interaction of DLGAP5 with METTL3 or NLRP3 was evaluated by Co-IP in primary hepatocytes and AML12 cells. **(G)** Protein levels of METTL3, DLGAP5, NLRP3, GSDMD-N and Cleaved CASPASE1 in primary hepatocytes transfected with si-*Mettl3*, si-*Dlgap5* or si-*Nlrp3* are determined. **(H)** PROGENy function profiling of NF-κB pathway in CCl_4_ group compared to control group.** (I)** GSEA enrichment plot and heatmap showing the enrichment of NF-κB pathway in CCl_4_ group compared to control group. **(J)** Correlation analysis of the expression of *Dlgap5* and NF-κB pathway in ALI.** (K)** Protein levels of NLRP3, ASC, Cleaved CASPASE1, GSDMD-N, mature IL-1β and mature IL-18 from indicated groups (n = 3 per group).** (L)** The relationship among the NLRP3, ASC and pro-CASPASE1 protein domains during the structural assembly process of NLRP3 inflammasome.** (M)** Interaction of NLRP3-PYD with ASC-PYD or ASC-CARD with Caspase1-CARD was evaluated by Co-IP in *Dlgap5*-overexpressed primary hepatocytes and control primary hepatocytes. All results are shown as mean ± SEM. *p < 0.05, **^#^**p < 0.05.

Next, we investigated whether METTL3 affected pyroptosis by regulation of DLGAP5. Initially, the protein levels of METTL3, DLGAP5 and NLRP3 in liver tissues and primary hepatocytes treated with CCl_4_ were elevated combinedly compared to the control group (Figure 5A-5B). IF staining revealed that the overlapped distribution of them, suggesting that DLGAP5 may interact with both of METTL3 and NLRP3 (Figure 5C). Further molecular docking showed the stable binding ability of DLGAP5 and METTL3 as well as NLRP3 through multiple sites (Figure 5D). Correlation analysis was conducted and revealed the positive relationship of *Mettl*3, *Dlgap*5 and *Nlrp3* expression in hepatocytes during ALI (Figure 5E). Co-IP assay further confirmed the interaction among them (Figure 5F). By separately or jointly transfection with si-*Mettl3*, si-*Dlgap5* or si-*Nlrp3* in hepatocytes, we found DLGAP5 level was controlled by METTL3 expression, and CCl_4_ treatment-mediated activation of GSDMD-N, Cleaved CASPASE1 and NLRP3 in pyroptotic hepatocytes were significantly inhibited by METTL3/DLGAP5/NLRP3 pathway silencing (Figure 5G). Current studies indicate that the NF-κB pathway serves as an essential priming signal for NLRP3 inflammasome activation 21-23. Transcriptome data of ALI mice treated with CCl_4_ was applied to evaluate the NF-κB pathway activity by PROGENy function profiling and GSEA analysis. The results showed that the NF-κB pathway was significantly activated with CCl_4_ treatment (Figure 5H-5I). Further correlation analysis between *Dlgap5* and the NF-κB pathway was conducted and revealed a positive relationship between them (Figure 5J). We further demonstrated that *Dlgap5* overexpression promoted NLRP3/CASPASE1 activation and GSDMD cleavage and pyroptosis, while these effects were abolished by the NF-κB inhibitor Bay117082 treatment (Figure 5K), establishing that DLGAP5 initiated NLRP3 inflammasome activation via NF-κB pathway. Furthermore, PYD domain-dependent NLRP3-ASC binding and CARD domain-mediated ASC-pro-Caspase1 binding are indispensable steps that driving the structural assembly of NLRP3 inflammasome (Figure 5L). Results of Co-IP experiments revealed that *Dlgap5* overexpression enhanced the interaction of NLRP3 and ASC in PYD domain and the binding of ASC and pro-CASPASE-1 in CARD domain, demonstrating the essential role of DLGAP5 in driving inflammasome formation and assembly through enhanced structural domain interactions (Figure 5M). Together, METTL3 elevates *Dlgap5* expression, and DLGAP5-driven NLRP3 inflammasome formation promoted pyroptosis, and silencing the METTL3/DLGAP5/NLRP3 pathway inhibits hepatocyte pyroptosis during ALI.

### Inhibition of DLGAP5 can effectively alleviate hepatocyte pyroptosis and acute liver injury

**Figure 6:**
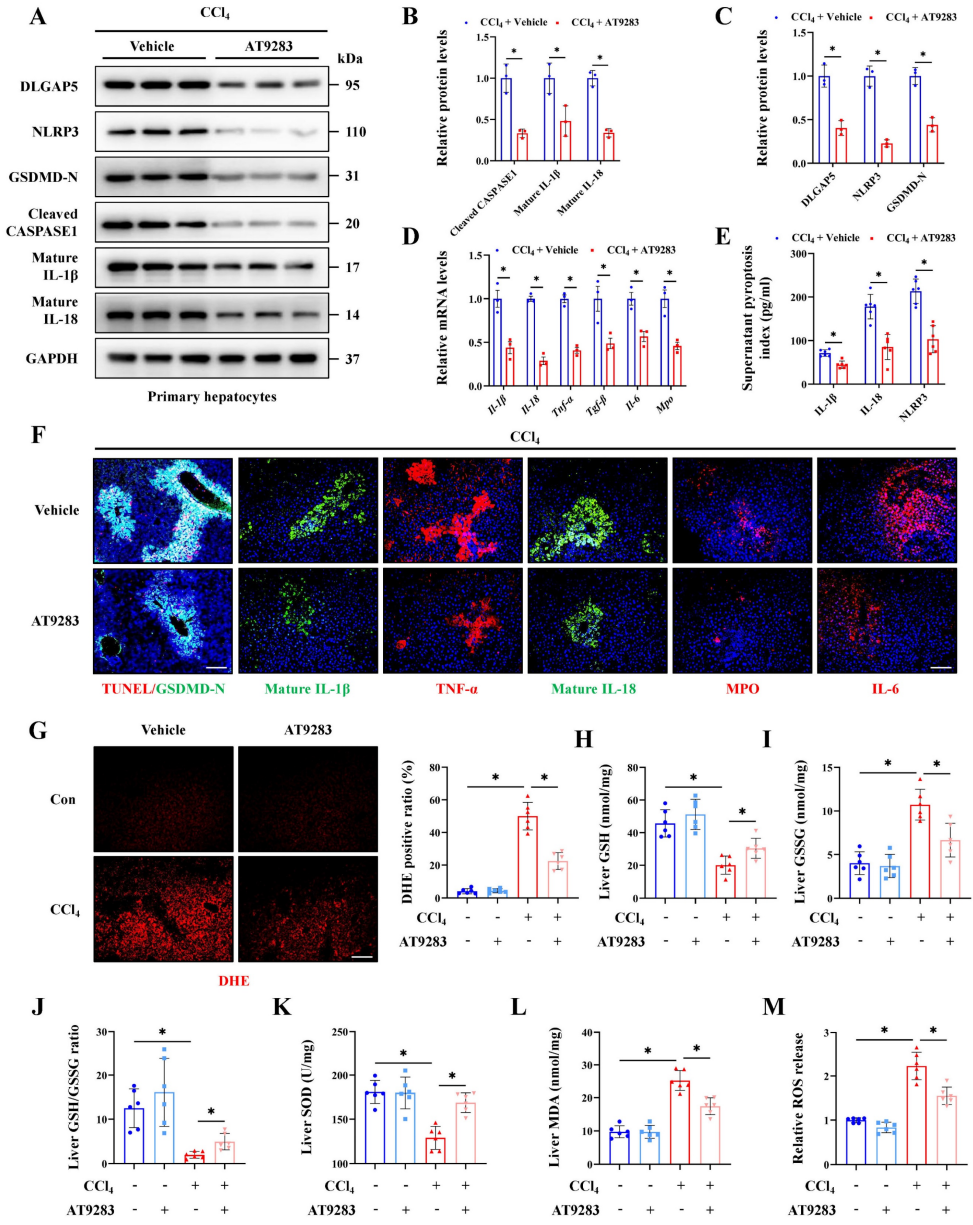
** Inhibition of DLGAP5 can effectively alleviate hepatocyte pyroptosis and acute liver injury. (A-C)** Protein expression of DLGAP5, NLRP3, GSDMD-N, Cleaved CASPASE1, mature IL-1β and mature IL-18 in primary hepatocytes was decreased under AT9283 treatment (n = 3 per group). **(D)** mRNA levels of *Il-1β*, *Il-18*, *Tnf-α*, *Tgf-β*, *Il-6* and *Mpo* in primary hepatocytes from indicated groups was determined by RT-qPCR (n = 3 per group).** (E)** Supernatant pyroptosis index from indicated groups was evaluated (n = 6 per group). **(F)** IF staining of mature IL-1β, mature IL-18, TNF-α, MPO, IL-6 (Scale bar: 50μm), together with TUNEL/GSDMD-N Co-IF (Scale bar: 25μm) in liver sections from indicated groups. **(G)** DHE staining in liver sections of above groups (Scale bar: 50μm, n = 6 per group). **(H-J)** Levels of liver GSH, GSSG and GSH/GSSG ratio from indicated groups (n = 6 per group). **(K, L)** Liver SOD and MDA level in indicated groups (n = 6 per group). **(M)** Relative ROS release content in above groups (n = 6 per group). All results are shown as mean ± SEM. *p < 0.05.

AT9283, a novel DLGAP5 inhibitor, and molecular docking showed that AT9283 can stably bind to DLGAP5 through multiple sites (Figure S4A). Further results indicated the expression of DLGAP5 in the group treated by AT9283 was significantly inhibited compared to control group, indicating the effectiveness of the inhibitor (Figure 6A, S4E). Meanwhile, the degree of hepatocyte inflammation, pyroptosis and liver injury were reduced with the inhibition of DLGAP5 (Figure 6A-6F, S4B-4H). Oxidative stress activated when antioxidant defense system imbalance, and the relationship of oxidative stress and pyroptosis was highlighted in acute organ injury. The results showed that CCl_4_ treatment significantly increased oxidative stress in the injured liver, manifested as an increase of DHE positive signal, MDA and ROS release, together with a decrease of GSH/GSSG ratio and SOD content. With the treatment of AT9283, the elevated oxidative stress indicators in CCl_4_-induced ALI models were alleviated (Figure 6G-6M). These results show that DLGAP5 plays a crucial role in hepatocyte pyroptosis and ALI, and DLGAP5 is an effective target in ALI.

### Hepatocyte pyroptosis activates macrophage M1 polarization to aggravate acute liver injury

**Figure 7:**
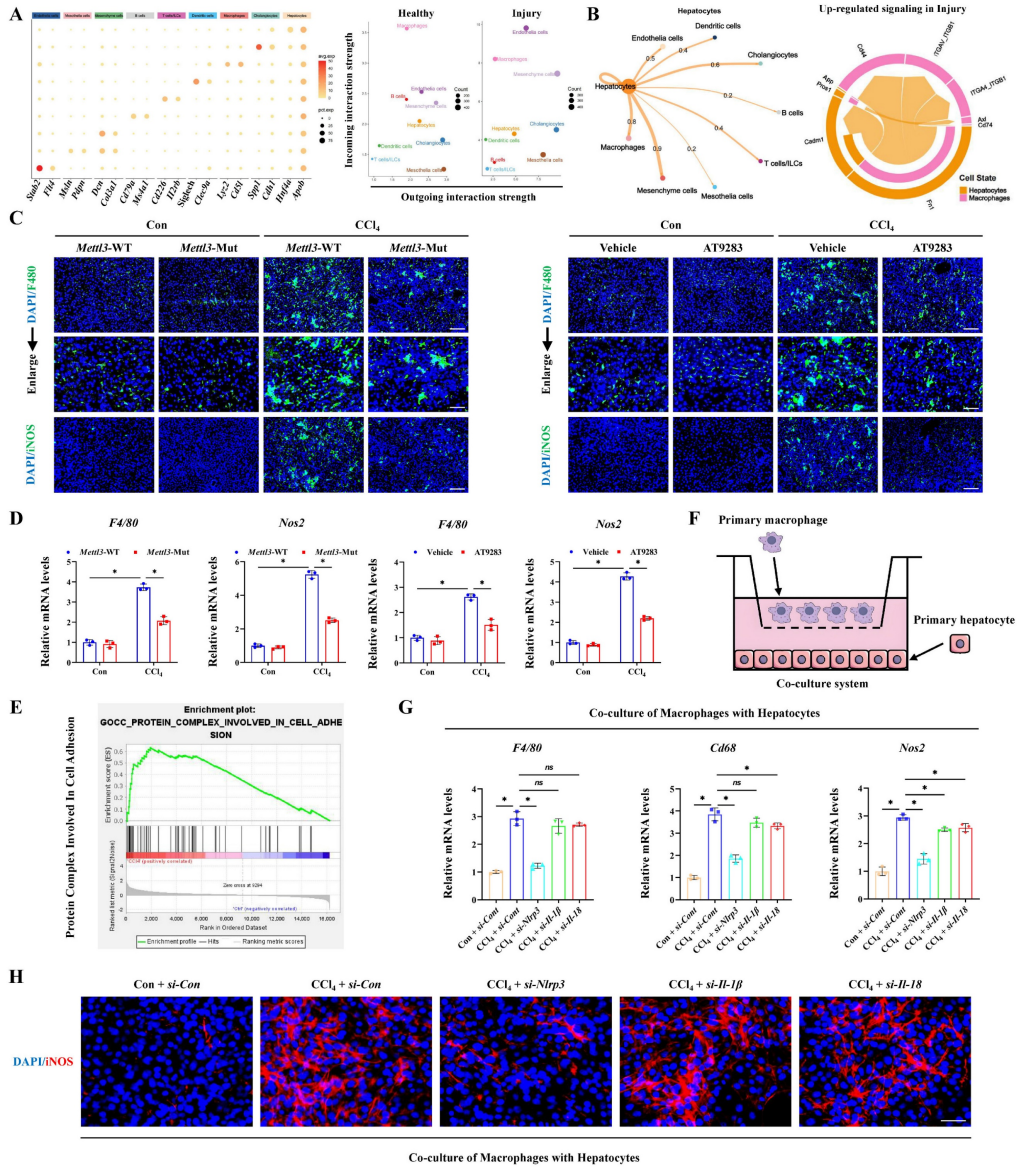
** Hepatocyte pyroptosis promotes macrophages M1 polarization to aggravate acute liver injury. (A)** Heatmap presented the expression of marker genes of indicated cell types. **(B)** Hepatocyte (source) interaction weights/strength in liver injury group (left panel) and the up-regulated signaling between hepatocytes and macrophages in liver injury group (right panel). **(C)** IF staining of F4/80 (Scale bar: 50μm, 25μm) and iNOS (Scale bar: 50μm) in liver sections from indicated groups. **(D)** mRNA levels of* F4/80* and *NOS2* from indicated groups (n = 3 per group). **(E)** GSEA analysis indicated the pathway of protein complex involved in cell adhesion was enriched in acute liver injury. **(F)** Schematic representation of co-culture model between primary hepatocytes and macrophages. **(G)** mRNA levels of *F4/80*, *Cd68* and *Nos2* in primary macrophages under co-culture with hepatocytes with different treatments (n = 3 per group). **(H)** iNOS immunofluorescence of primary macrophages by co-culture with hepatocytes with different treatments. All results are shown as mean ± SEM. *p < 0.05. ns, not significant.

According to the RNA-seq results from CCl_4_-induced ALI models, GSEA analysis showed the pathway-related to cell-cell adhesion, cell substrate adhesion, cell substrate junction and cell-cell recognition were significantly enriched, indicating that pyroptotic hepatocyte may further communicated with other types of liver cells in ALI (Figure S5A). GO assay further revealed a number of macrophage-related pathways were activated, suggesting the potential interaction of macrophages and pyroptotic hepatocytes in ALI (Figure S5B). Next, single-nucleus RNA sequencing (sn-RNA-seq) data of ALI mice was applied to investigate the potential communications between hepatocytes and other types of liver cells deeply. We firstly identified and visualized 9 clusters using the Uniform Manifold Approximation and Projection (UMAP) method (Figure S5C), and found significant cell population alterations in the liver after ALI (Figure S5D). Heatmap showed the expression of marker genes in the indicated cell types (Figure 7A). Consistent with the enrichment results of RNA-seq, cell-chat analysis of sn-RNA-seq verified that macrophages most closely interacted with hepatocytes in liver injury (Figure 7B). M1-type macrophage is a pro-inflammatory phenotype of activated macrophage, involving in various inflammatory diseases. Our results showed the expression of M1-type macrophage specific marker iNOS increased after CCl_4_ treatment, indicating M1 polarization of macrophages (Figure 7C, 7D). More importantly, accompanied with the alleviated liver injury, M1 polarization of macrophages was also rescued by *Mettl3* mutation and DLGAP5 inhibition (by AT9283), which may be mediated by hepatocyte pyroptosis blockade (Figure 7C, 7D).

Further, enrichment analysis suggested that hepatocyte-macrophage interaction may be mediated through the secretion of protein complexes (Figure 7E). And co-culture system was established to explore the direct role of hepatocyte pyroptosis on macrophage M1 polarization (Figure 7F). As the key effectors of pyroptosis, hepatocyte-derived NLRP3, IL-1β and IL-18 was respectively focused to evaluate the contributes of hepatocyte injury on macrophage activity. Results showed that inhibition of the IL-1β or IL-18 expression in hepatocytes did not significantly downregulate macrophage M1 polarization induced by pyroptotic hepatocytes from CCl_4_-treated mice in the co-culture system, while inhibition of NLRP3 significantly reduced that (Figure 7G, 7H). However, existing studies reported that IL-1β and IL-18 can induce macrophage M1 polarization 24-26. Given this apparent inconsistency, we hypothesized that subthreshold interleukin concentrations within the co-culture microenvironment might underlie the unresponsiveness to IL-18/IL-1β inhibition. To verify that, we quantified IL-18 and IL-1β levels in the co-culture system containing primary hepatocytes (2×10⁵ cells) and primary macrophages. The results showed that both IL-18 and IL-1β levels were approximately 100 pg/mL (Figure S6A). According to past reports, the concentration gradients of IL-1β and IL-18 used to stimulate M1 polarization of macrophages ranged from 1-50 ng/mL 24, 25, which was much higher than the concentration detected in our co-culture system. To accurately explore the role of IL-1β and IL-18 in M1 polarization of macrophages within our study, exogenous IL-1β (10 ng/mL) or IL-18 (20 ng/mL) was added to the co-culture system. The results demonstrated that sufficient supplement of either IL-1β or IL-18 further promoted macrophages M1 polarization by co-culture with primary hepatocytes from CCl_4_-treated mice (Figure S6B, S6C). In addition, macrophage M1 polarization suppressed by hepatocyte *Nlrp3* knockdown in co-culture system was restored by adequate exogenous IL-1β (10 ng/mL) or IL-18 (20 ng/mL) addition (Figure S6D, S6E). Together, these findings suggest that macrophage M1 polarization can be activated by pyroptotic hepatocyte to aggravate ALI.

### Inhibition of hepatocyte pyroptosis alleviates acute liver injury by blocking macrophage M1 polarization and metabolic reprogramming

**Figure 8:**
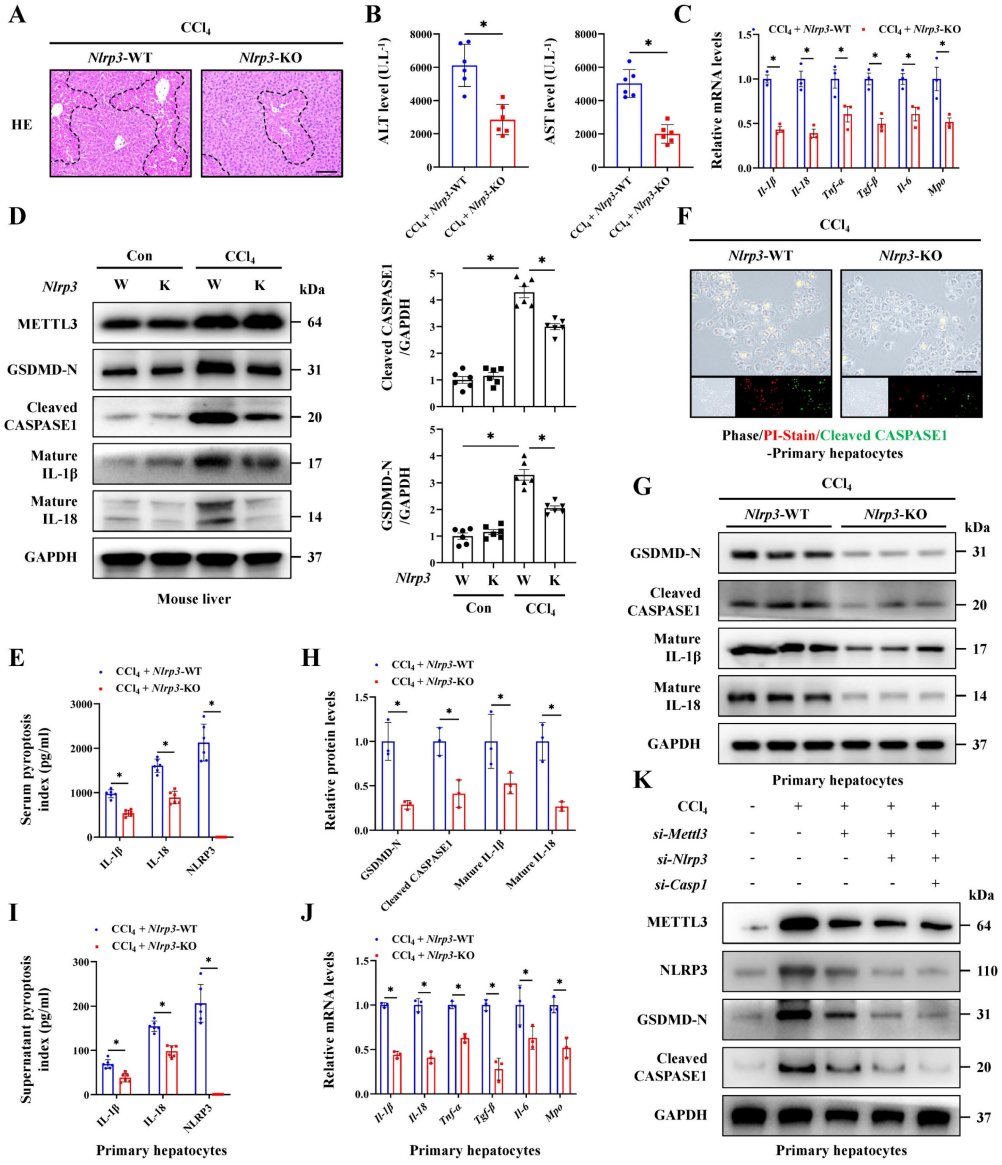
** Inhibition of NLRP3 alleviates acute liver injury by reducing hepatocyte pyroptosis. (A)** H&E staining in liver sections of NLRP3-WT and NLRP3-KO mice under CCl_4_ treatment (Scale bar: 50μm). **(B)** Serum ALT and AST levels from indicated groups (n = 6 per group). **(C)** mRNA levels of *Il-1β*, *Il-18*, *Tnf-α*, *Tgf-β*, *Il-6* and *Mpo* in liver tissues of above groups (n = 3 per group). **(D)** Protein levels of METTL3, GSDMD-N, Cleaved CASPASE1, mature IL-1β and mature IL-18 in mouse livers from indicated group (n = 6 per group). **(E)** Serum pyroptosis index in indicated groups (n = 6 per group). **(F)** PI/Cleaved CASPASE1 co-staining in primary hepatocytes from NLRP3-WT and NLRP3-KO mice under CCl_4_ treatment (Scale bar: 25μm). **(G, H)** Protein expression of GSDMD-N, Cleaved CASPASE1, mature IL-1β and mature IL-18 in primary hepatocytes from indicated group (n = 3 per group). **(I)** Supernatant pyroptosis index of primary hepatocytes from indicated groups (n = 6 per group).** (J)** mRNA levels of *Il-1β*, *Il-18*, *Tnf-α*, *Tgf-β*, *Il-6* and *Mpo* in primary hepatocytes from indicated groups (n = 3 per group). **(K)** Protein levels of METTL3, NLRP3, GSDMD-N and Cleaved CASPASE1 in primary hepatocytes transfected with si-*Mettl3*, si-*Nlrp3* or si-*Caspase1* from control or CCl_4_-treated mice. All results are shown as mean ± SEM. *p < 0.05.

**Figure 9:**
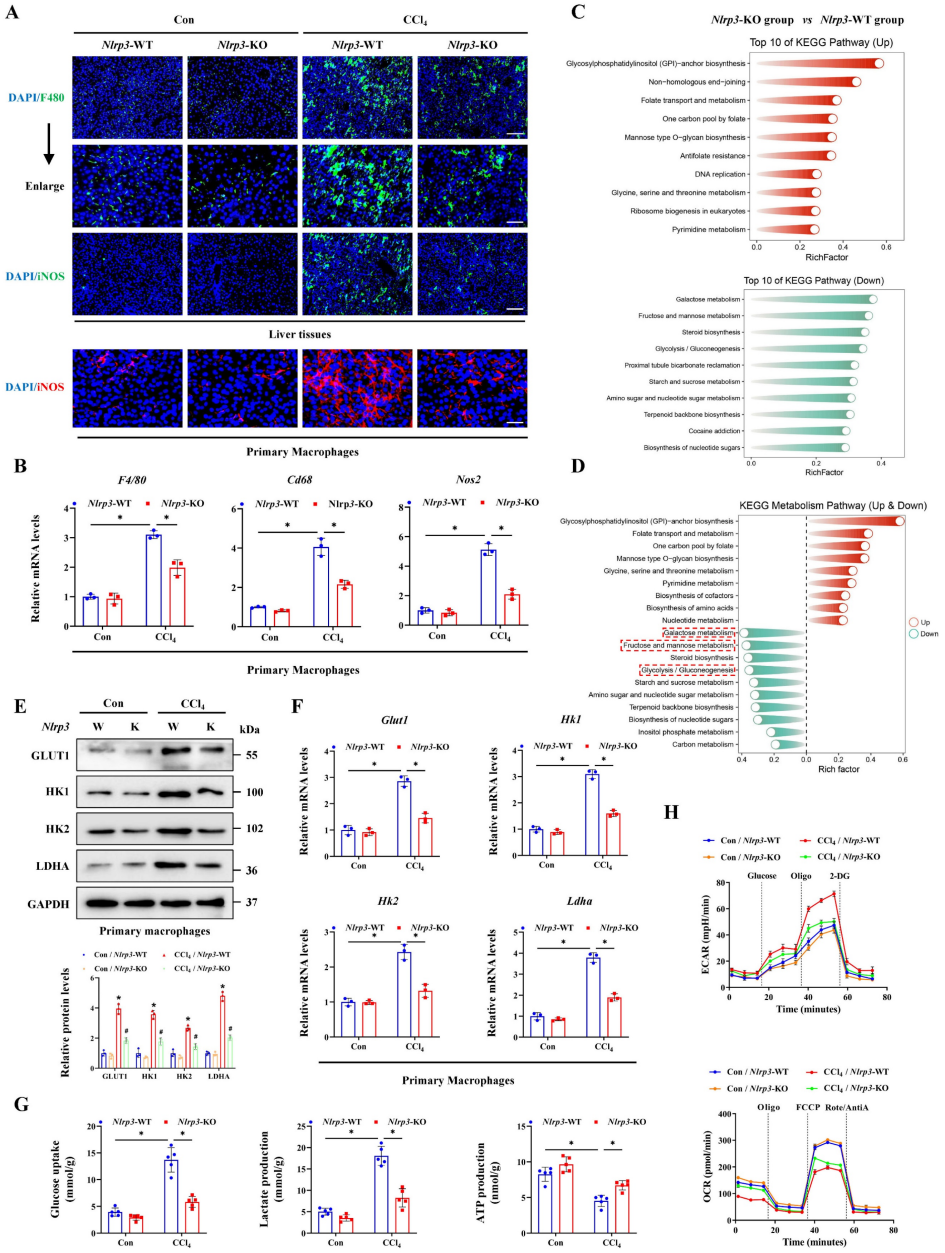
** Inhibition of NLRP3 blocks macrophage M1 polarization and metabolic reprogramming. (A)** IF staining of F4/80 (Scale bar: 50μm, 25μm) and iNOS (Scale bar: 50μm) in liver sections of *Nlrp3*-WT and *Nlrp3*-KO mice treated with or without CCl_4_. And iNOS fluorescence in primary macrophages from above groups (Scale bar: 25μm).** (B)** mRNA levels of *F4/80*, *Cd68* and *Nos2* in primary macrophages from indicated group (n = 3 per group). **(C)** KEGG analysis of DEGs generated from primary macrophages of *Nlrp3*-KO and *Nlrp3*-WT mice under CCl_4_ treatment. **(D)** Top 10 up-regulated and down-regulated pathways related to metabolism enriched in *Nlrp3*-KO group compared to *Nlrp3*-WT group.** (E)** mRNA levels of *Glut1*, *Hk1*, *Hk2* and *Ldha* in primary macrophages from indicated group (n = 3 per group). **(F)** Protein expression of GLUT1, HK1, HK2 and LDHA in primary macrophages from above groups (n = 3 per group). **(G)** Glucose uptake, lactate production and ATP production in primary macrophages from indicated group (n = 5 per group). **(H)** Measurement of ECAR and OCR from above groups. All results are shown as mean ± SEM. *p < 0.05, **^#^**p < 0.05.

Basing the critical role of NLRP3 in interleukin release and the pyroptosis pathway activation, together with the involvement in hepatocyte-macrophage interaction for inducing ALI, we further constructed the *Nlrp3* knockout (*Nlrp3*-KO) mice. Firstly, the degree of liver injury was significantly reduced by *Nlrp3* knockout (Figure 8A, 8B), and the pyroptosis pathway was also inhibited in liver tissues and primary hepatocytes from *Nlrp3*-KO mice compared with *Nlrp3*-WT mice (Figure 8C-8J). Meanwhile, si-RNA treatment further confirmed the role of METTL3-m6A/NLRP3 signaling in pyroptosis regulation (Figure 8K). We next discussed the effects of hepatocyte pyroptosis induced by NLRP3 on macrophages, and the results from total liver tissues and primary macrophages indicated that macrophage M1 polarization in *Nlrp3*-KO mice treated with CCl_4_ was reduced compared to wild type mice (Figure 9A, 9B). These data indicated that inhibition of *Nlrp3* can not only alleviate ALI by blocking the pyroptotic pathway, but also by blocking communication between macrophage and hepatocyte. To further investigate the potential mechanism of macrophage M1 polarization, transcriptome sequencing was performed on primary macrophages isolated from the livers of *Nlrp3*-KO and *Nlrp3*-WT mice under CCl_4_ treatment. KEGG analysis revealed the significant enrichment of metabolism-related pathways in macrophages during ALI (Figure 9C). Further metabolism pathway analysis identified galactose metabolism, fructose and mannose metabolism, steroid biosynthesis and glycolysis were the most significantly downregulated pathways, while glycosylphosphatidylinositol (GPI)-anchor biosynthesis, folate transport and metabolism, and one carbon pool by folate were the most markedly upregulated pathways in *Nlrp3*-KO group compare to *Nlrp3*-WT group (Figure 9D). The heatmap of genes within these pathways was presented in Figure S7A. We further found that the metabolic pathways related to glycolysis was significantly inhibited in *Nlrp3*-KO mice, which is a key metabolic feature of macrophage M1 polarization (Figure 9D). The results of RT-qPCR and WB demonstrated that the expression of key glycolytic regulators were increased under CCl_4_ treatment, while significantly reduced in *Nlrp3*-KO mice compared to WT group (Figure 9E, 9F). Glycolytic flux Assays further confirmed the attenuated glycolytic flux in *Nlrp3*-KO mice, evidenced by decreased glucose uptake and lactate production, alongside increased ATP levels (Figure 9G). Real-time metabolic profiling via seahorse assay corroborated this blunted glycolytic capacity with *Nlrp3* inhibition, showing significant reductions in both basal extracellular acidification rate (ECAR) and maximal glycolytic flux. Conversely, oxygen consumption rate (OCR) was elevated, indicating a compensatory shift toward oxidative phosphorylation in *Nlrp3*-KO mice (Figure 9H).

Finally, to verify that macrophage alterations in metabolic reprogramming and M1 polarization were specifically driven by pyroptotic hepatocytes, primary hepatocytes isolated from Con- or CCl_4_-treated wild-type mice and *Nlrp3*-KO mice were co-cultured with primary normal macrophages. The results demonstrated that hepatocytes from CCl_4_-injured WT mice significantly enhanced glycolysis and M1 polarization of macrophages compared to hepatocytes from control mice, while the absence of *Nlrp3* in hepatocytes reversed the effects (Figure S7B-S7G). These results suggest that pyroptotic hepatocytes mediated by NLRP3 may drive macrophage metabolic reprogramming and M1 polarization, thereby activate the pro-inflammatory phenotype of macrophages to exacerbates ALI. In conclusion, METTL3 catalyzes the m6A methylation of *Dlgap5* with the assistance of IGF2BP2, thereby enhancing the activation and formation of NLRP3 inflammasome which initiating the pyroptosis pathway. The hepatocyte pyroptosis not only induce hepatocyte death directly, but also promote metabolic reprogramming and M1 polarization of macrophages through intercellular communication, which leads to the worsening of ALI.

## Discussion

ALI is a global public health issue and the limited treatment option is the key reason for its development into liver failure and death 27. Up to now, the exact mechanism of ALI has still not been fully elucidated and there is an urgent need to find new effective intervention targets. The massive death of hepatocytes and the subsequent severe inflammatory response are the common characteristics in ALI, regardless of the initial trigger 28. Pyroptosis is a novel inflammatory cell death modality that differs from other types of cell death in both morphology and mechanism 29. Current research suggests that NLRP3 activated by various internal and external factors can induce self-cleavage and activation of CASPASE1, and the active N-terminal region of GSDMD cleaved by the activated CASPASE1 will insert into the cell membrane and perforate, leading to cell swelling and pyroptosis.

At the same time, the pro-IL-1β and pro-IL-18 cleaved by CASPASE1 were exchanged into mature IL-1β and IL-18 and released through the aforementioned pores to trigger further inflammatory reactions 2, 30, 31. Pyroptosis is a key regulatory factor in inflammatory diseases, while research regarding pyroptosis in the field of liver disease mainly focuses on chronic stages such as fibrosis, cancer, etc. The role of pyroptosis in ALI has not been widely studied. In this study, pyroptosis was determined in ALI. In CCl_4_-and TAA- induced mouse ALI models, injured hepatocyte swelled and fragmented, and the activated NLRP3, GSDMD-N and Cleaved CASPASE1 promoted the release of IL-1β and IL-18. With the extension of CCl_4_ or TAA treatment, LDH activity and pyroptotic hepatocytes increased continuously, indicating that the classic pyroptosis of hepatocytes in ALI. Therefore, we indicate that pyroptosis may be a commonly activated cell death pathway during the progression of acute liver injury, rather than being a feature specific to damage caused by a particular compound, and pyroptosis is involved in the whole stage of liver diseases.

m6A modification is an important phenomenon in epigenetics and has been shown to dynamically and reversibly exert biological functions through various pathways, including affecting splicing, output, degradation, and translation initiation efficiency, to regulate mRNA fate 32-34. As a core m6A methyltransferase, METTL3 mediates the m6A methylation process in liver diseases. A study found that upregulation of STING mediated by METTL3 is one of the causes of radiation-induced liver injury 35; and two other studies reported that downregulation of METTL3 was involved in both cadmium and acetaminophen induced liver injury 36, 37. The role of METTL3-mediated m6A modification in ALI was controversial and the specific function and mechanism need further exploration. Here, we found m6A methylation was significantly elevated in acute liver injury and primarily catalyzed by METTL3. Inhibition of METTL3-mediated m6A process alleviated hepatocyte pyroptosis in ALI. By further MeRIP-seq and RNA-seq data, we preliminarily screened *Dlgap5* as the target in METTL3-mediated m6A methylation. DLGAP5, also known as DLG7 or HURP, is a member of the DLGAP protein family and located on chromosome 14q22.3, and is a microtubule associated protein encoded by a gene that regulates the cell cycle 38. As a Ran GTPase effector, DLGAP5 participates in the stable regulation of mitotic centromere fibers, microtubule aggregation, bipolar spindle formation and capture of kinetochore fibers 39, 40. Past research revealed that DLGAP5 is mainly related to various types of cancers, including breast cancer, bladder cancer, gastric cancer, etc 41-43. Here, we also found that DLGAP5 expression was upregulated in both liver tissue and primary hepatocytes of ALI, highlighting the role of DLGAP5 in acute inflammatory liver diseases. As reported, the expression of DLGAP5 can also be upregulated by m6A methylation 44. Subsequently, we further discovered that the *Dlgap5* m6A modification was recognized by the m6A reader IGF2BP2 in hepatocytes. RIP assay confirmed that DLGAP5 can directly bind to IGF2BP2, which promoted *Dlgap5* mRNA translation to induce ALI. Then we found that the expression of *Dlgap5* is positively correlated with *Metll3* and *Nlrp3* expression respectively, and DLGAP5 could interact with METTL3 and NLRP3. Deeply, we demonstrated that DLGAP5 drove inflammasome formation via a dual-axis mechanism, where DLGAP5 initiated NLRP3 inflammasome activation via NF-κB pathway, then facilitated inflammasome formation and assembly through enhancing the interaction of NLRP3 and ASC in PYD domain and the binding of ASC and pro-CASPASE-1 in CARD domain, indicating that DLGAP5 acted as a scaffold protein facilitating domain-specific oligomerization. By step-by-step experiments, we demonstrate that DLGAP5 serves as a bridge between m6A methylation and hepatocyte pyroptosis, which was an inducer of NLRP3 inflammasome formation and subsequent hepatocyte pyroptosis.

Notably, we noticed that METTL3 and DLGAP5 are not completely co-located in subcellular distribution, suggesting that the possibility of additional regulatory mechanisms beyond METTL3 that control *Dlgap5* expression during acute liver injury. Hepatic regenerative signaling pathways are concurrently activated in the context of acute liver injury, and core pathways, including HGF/c-Met, Wnt/β-catenin and IL-6/STAT3, drive hepatocytes into the cell cycle and promote proliferation and hepatic regeneration 45, 46. Given the established role of DLGAP5 as a core regulator of the cell cycle, we hypothesize that these pro-regenerative signaling pathways themselves may also constitute significant upstream regulators of *Dlgap5* expression in acute liver injury. Additionally, non-coding RNA (ncRNA) are widely recognized as key regulators in the pathogenesis of acute liver injury, modulating various biological processes such as inflammation, oxidative stress, and hepatocyte death, which have also been reported to directly or indirectly regulate the expression of DLGAP5 within the process 47, 48. Likewise, DLGAP5 and NLRP3 also exhibited incomplete subcellular co-localization, suggesting that beyond mediating pyroptosis, DLGAP5 may regulate additional cellular processes. As mentioned above, DLGAP5 may promote liver regeneration in ALI by driving cell cycle progression; meanwhile, existing study also indicate the anti-apoptotic regulatory function of DLGAP5 49, suggesting its potential multiple effects in hepatic biology: beyond participating in ALI via pyroptosis, may also contribute to hepatic protection and repair mechanisms in a certain extent. Collectively, *Dlgap5* expression may be influenced by multiple regulatory mechanisms beyond METTL3-mediated m6A modification and may cause various effects beyond the involvement in pyroptosis. Future studies needed to reveal the comprehensive roles and regulatory factors of DLGAP5 in liver injury.

Compared to other parenchymal organs, macrophages account for the highest proportion of total immune cells in the liver. In healthy rodent livers, every 100 hepatocytes are accompanied by 20-40 macrophages 50. These large numbers of macrophages play crucial roles in maintaining liver tissue homeostasis and inducing inflammatory liver diseases. During ALI, macrophages sense the damage to the liver and trigger its activation, subsequently releasing inflammatory chemokines and cytokines and recruiting circular monocytes to infiltrate the liver and thereby transdifferentiating into macrophages for injury maintenance 51. The expanding pool of macrophages actively engages in bidirectional communication with damaged hepatocytes, a crucial process that drives pro-inflammatory (M1) polarization and amplifies liver injury 52, 53. In this study, we revealed the close intercellular communication between pyroptotic hepatocytes and macrophages, and then verified macrophage M1 polarization in ALI. As the key effectors of pyroptosis, we showed that inhibition of NLRP3 in hepatocytes significantly reduced macrophage M1 polarization induced by pyroptotic hepatocytes from ALI mice in the co-culture system, while inhibition of the IL-1β or IL-18 expression did not significantly downregulate that. These were contradictory with established literatures regarding that IL-1β and IL-18 promoted macrophage M1 polarization to some extent 24, 25. Basing that, we assumed this discrepancy stemmed from the inability of co-culture system *in vitro* to fully simulate *in vivo* cellular communication dynamics, and which may be caused by the low concentration of interleukins from original pyroptotic hepatocytes in the co-culture system, which was insufficient to drive M1 polarization of macrophages. Therefore, macrophage polarization phenotype changes did not appear after interleukin knockdown in hepatocytes. Strikingly, quantitative analysis of IL-18 and IL-1β levels in the co-culture system revealed that the concentration of both interleukins was significantly lower than the lowest concentration to be capable of promoting macrophage M1 polarization reported in the literature 24, 25. Supplementation with sufficient exogenous IL-1β and IL-18 not only further enhanced macrophages M1 polarization by co-culture with primary hepatocytes from ALI mice, but also rescued the suppressed M1 polarization phenotype resulting from co-culture with NLRP3-deficient hepatocyte. Due to the sustained interleukin secretion by hepatocytes and continuous crosstalk between hepatocytes and macrophages *in vivo*, the cumulative interleukin levels profoundly shape macrophage polarization. These results indicate that all of NLRP3 inflammasome, IL-1β and IL-18 released by pyroptotic hepatocyte can effectively promote macrophage M1 polarization.

Metabolic reprogramming dynamically balances distinct metabolic pathways, playing a decisive role in polarizing macrophages toward either pro-inflammatory (M1) or anti-inflammatory (M2) phenotypes 54, 55. Critically, enhanced glycolysis, a hallmark metabolic shift in M1-polarized macrophages, not only fuels pro-inflammatory responses through rapid ATP generation and lactate accumulation, but also amplifies liver injury by sustaining macrophage-derived cytokine storms and hepatocyte death 56-58. Given the core role of NLRP3 in the pyroptosis pathway and its involvement in hepatocyte-macrophage interaction during ALI, *Nlrp3*-KO mice were further constructed. Transcriptomic analysis from primary macrophages showed metabolic reprogramming toward glycolysis was inhibited by *Nlrp3* deficiency. And hepatocyte pyroptosis may drive metabolic reprogramming in macrophages. Through expression profiling of key glycolytic genes, glycolytic flux assessment, and real-time metabolic profiling via Seahorse assay, we demonstrated that macrophages rapidly shift from a quiescent, mitochondrial oxidative phosphorylation-dependent state to a glycolysis-dominant metabolic mode under pyroptotic hepatocyte stimulation. This metabolic shift aligns with established metabolic signatures known to drive M1 polarization, revealing the specific mechanism of macrophage M1 polarization and metabolic reprogramming promoted by pyroptotic hepatocytes. The activated pro-inflammatory macrophages secrete various cytokines such as TNF-α, IFN-γ, MCP-1, CXCL-10, which further induce hepatocyte death and recruit more immune cells to exacerbate liver damage 59. While, inhibition of the infiltration and activation of macrophages exhibit a protective effect in acute liver injury. Mossanen et al. 60 found that CCR-2 antagonists-mediated reduction of bone marrow-derived macrophages infiltration can improve acute liver injury induced by acetaminophen. Schümann et al. 61 also revealed that liver injury is limited to a few small necrotic areas after removing macrophages, and GdCl3 showed potential liver protective effects in liver ischemia-reperfusion injury by inhibiting early activation of macrophages 62. Together, *Nlrp3* deficiency reduces hepatocyte pyroptosis, and further inhibits macrophage metabolic reprogramming and M1 polarization to rescue hepatocyte-macrophage communication for alleviating acute liver injury.

In conclusion, our study demonstrates that METTL3-induced m6A methylation plays an important role in the process of ALI by promoting hepatocyte pyroptosis and disrupting communication between hepatocytes and macrophages. *Dlgap5* serves as the target of METTL3-induced m6A methylation and induces hepatocyte pyroptosis by promoting NLRP3 inflammasome activation and formation. Subsequently, pyroptosis products releasing by the GSDMD-N-dependent membrane pores from hepatocytes activated macrophages metabolic reprogramming and M1 polarization to further exacerbate ALI. These findings reveal the potential mechanisms of ALI from an intercellular communication perspective, and targeted -inhibition of DLGAP5 and -blockade of hepatocyte-macrophage interaction provide promising strategies for ALI treatment.

## Supplementary Material

Supplementary figures and tables.

## Abbreviations

ALI: acute liver injury
ALT: alanine aminotransferase
AST: aspartate aminotransferase
CCl_4_: carbon tetrachloride
Co-IP: co-immunoprecipitation
DAB: Diaminobenzidine
DAMPs: damage associated molecular patterns
ECAR: extracellular acidification rate
ELISA: enzyme linked immunosorbent assay
FBS: fetal bovine serum
GO: gene ontology
GSH: glutathione
GSSG: oxidized glutathione disulfide
H&E: hematoxylin and eosin
HSC: hepatic stellate cell
IF: Immunofluorescence
IHC: Immunohistochemistry
IL: Interleukin
iNOS: inducible nitric oxide synthase
KEGG: Kyoto Encyclopedia of Genes and Genomes
KO: knockout
M6A: N6-methyladenosine
MDA: malondialdehyde
MeRIP: methylated RNA Immunoprecipitation
METTL3: methyltransferase-like 3
Mut: Mutant
NLRP3: NOD-like receptor family pyrin domain containing 3
NPC: non-parenchymal cell
OCR: oxygen consumption rate
PBS: phosphate buffered saline
PCR: polymerase chain reaction
PVDF: polyvinylidene fluoride
RIP: RNA immunoprecipitation
RNA-seq: RNA sequencing
ROS: reactive oxygen species
RT-qPCR: real-time quantitative polymerase chain reaction
siRNA: small interfering RNA
SOD: superoxide dismutase
TAA: thioacetamide
TUNEL: terminal deoxynucleotidyl transferase dUTP nick end labeling
WB: western blot
WT: wildtype
