# Supplementary material for: DLGAP5 Promotes Acute Liver Injury via Hepatocyte Pyroptosis-Driven Macrophage Metabolic Reprogramming and M1 Polarization
Source: Int J Biol Sci. 2025 Aug 30;21(12):5563–85. doi: 10.7150/ijbs.118024 (PMC12435577; doi:10.7150/ijbs.118024)
Supplement: Supplementary file 1 — Supplementary figures and tables. [file ijbsv21p5563s1.pdf]

Figure S1. Pyroptosis is involved in acute liver injury induced by TAA. (A) H&E staining in liver sections (Scale bar: 50μm, 25μm) and serum ALT and AST levels of control group and TAA group (n = 6 per group). **(B)** IHC staining of GSDMD-N and Cleaved CASPASE1 in liver sections from indicated groups (Scale bar: 50μm, n = 6 per group). **(C)** Protein expression of GSDMD-N, Cleaved CASPASE1, mature IL-1β and mature IL-18 in mouse livers treated with PBS or TAA (n = 3 per group). **(D)** Ultrastructural features identified by TEM under control and TAA treatment (Scale bar: 5μm, 2μm, n = 6 per group). (**E, F**) TUNEL/GSDMD-N co-staining (Scale bar: 25μm) and IF staining of mature IL-1β and mature IL-18 (Scale bar: 50μm) in liver sections from indicated groups (n = 6 per group). **(G)** mRNA levels of *Il-1β*, *Il-18*, *Tnf-α*, *Tgf-β*, *Il-6* and *Mpo* in liver tissues were determined by RT-qPCR (n = 3 per group). **(H)** The levels of CASPASE1 activity, IL-1 and IL-18 in serum from indicated group were measured (n = 6 per group). **(I)** Phase contrast micrographs of primary hepatocytes from mice treated with TAA at 0, 6, 12, 24, and 48 hours (Scale bar: 50μm). **(J)** LDH activity in the cell culture supernatant at different timeline from indicated group (n = 3 per group). **(K)** PI/Cleaved CASPASE1 co-staining of primary hepatocytes at different timeline of TAA treatment (Scale bar: 50μm, n = 3 per group). All results are shown as mean ± SEM. *$p < 0.05$.

Figure S2. Hepatocyte pyroptosis is involved in acute liver injury. (A) Signaling pathways related to inflammation and cell death were enriched by GO analysis in the primary hepatocytes from CCl_4-treated mice. **(B, C)** Protein levels of GSDMD-N, Cleaved CASPASE1, mature IL-1β and mature IL-18 in primary hepatocytes under control and CCl_4 treatment (n = 3 per group). **(D)** mRNA levels of *Il-1β*, *Il-18*, *Tnf-α*, *Tgf-β*, *Il-6* and *Mpo* in primary hepatocytes from indicated groups (n = 3 per group). **(E)** CASPASE1 activity and the IL-1β and IL-18 level in the cell culture supernatant (n = 6 per group). All results are shown as mean ± SEM. *$p < 0.05$.

Figure S3. Hepatocyte pyroptosis is regulated by METTL3. (A) DNA gel electrophoresis confirming successful construction of the *Mettl3*-Mut mice. **(B)** Protein expression of METTL3 in liver tissues and primary hepatocytes from *Mettl3*-WT and *Mettl3*-Mut control mice (n = 3 per group). **(C)** mRNA level of *Mettl3* in liver tissues and primary hepatocytes from *Mettl3*-WT and *Mettl3*-Mut control mice (n = 3 per group). **(D)** Protein expression of METTL3 and NLRP3 in primary hepatocytes. **(E)** Protein levels of METTL3, NLRP3, GSDMD-N, Cleaved CASPASE1, mature IL-1β and mature IL-18 in primary hepatocytes isolated from *Mettl3*-WT and *Mettl3*-Mut mice under CCl_4 treatment (n = 3 per group). **(F)** mRNA levels of *Mettl3*, *Il-1β*, *Il-18*, *Tnf-α*, *Tgf-β*, *Il-6* and *Mpo* in primary hepatocytes from indicated groups (n = 3 per group). **(G)**

Supernatant pyroptosis index of primary hepatocytes in indicated groups (n = 6 per group). **(H)** LDH activity in the culture supernatant of primary hepatocytes at different timeline (n = 6 per group). **(I)** Protein levels of METTL3, NLRP3, GSDMD-N and Cleaved CASPASE1 in primary hepatocytes transfected with si-*Mettl3* or control siRNA from control or CCl_4-treated mice (n = 3 per group). All results are shown as mean ± SEM. *$p < 0.05$. ns, not significant.

Figure S4. Inhibition of DLGAP5 can effectively alleviate pyroptosis and acute liver injury. (A) Molecular docking of AT9283 to DLGAP5 protein. **(B)** H&E staining in liver sections of ALI mice with or without AT9283 treatment (Scale bar: 50μm). **(C)** Serum ALT and AST levels from indicated groups (n = 6 per group). **(D)** mRNA levels of *Il-1β*, *Il-18*, *Tnf-α*, *Tgf-β*, *Il-6* and *Mpo* in liver tissues of above groups (n = 3 per group). **(E)** AT9283 treatment reduced the protein levels of DLGAP5, NLRP3, METTL3, GSDMD-N, Cleaved CASPASE1, mature IL-1β and mature IL-18 in ALI mouse liver (n = 6 per group). **(F, G)** IHC staining of GSDMD-N, Cleaved CASPASE1 and NLRP3 in liver sections from indicated groups (Scale bar: 50μm, n = 6 per group). **(H)** Serum pyroptosis index from indicated groups (n = 6 per group). All results are shown as mean ± SEM. *$p < 0.05$. ns, not significant.

Figure S5. CCl_4 treatment affects cellular communication in the liver. (A) Signaling pathways related to cell communication were enriched according to the gene set enrichment analysis (GSEA). **(B)** Signaling pathways related to macrophage activation was screened by the GO analysis. **(C)** Cell clusters were identified and visualized by sn-RNA-seq. **(D)** The bar chart showed the proportion of cell types from healthy group and injury group.

Figure S6. Sufficient IL-1β or IL-18 signaling drives macrophage M1 polarization. (A) Concentration of IL-1β and IL-18 in the supernatant of primary hepatocytes for co-culture (n = 6 per group) **(B)** iNOS fluorescence showing that sufficient supplement of either IL-1β (10 ng/ml) or IL-18 (20 ng/ml) further promoted macrophages M1 polarization by co-culture with primary hepatocytes from CCl_4-treated mice (Scale bar: 25μm). **(C)** mRNA levels of *F4/80*, *Cd68* and *Nos2* in macrophages after PBS, IL-1β or IL-18 addition to the above co-culture system (n = 3 per group). **(D)** mRNA levels of *F4/80*, *Cd68* and *Nos2* in macrophages demonstrated that macrophage M1 polarization suppressed by hepatocyte *Nlrp3* knockdown in co-culture system was restored by adequate exogenous IL-1β (10 ng/ml) or IL-18 (20 ng/ml) addition (n = 3 per group) **(E)** iNOS fluorescence revealed macrophage M1 polarization suppressed by hepatocyte *Nlrp3*

knockdown in co-culture system was restored by adequate exogenous IL-1β or IL-18 addition. All results are shown as mean ± SEM. $^{*}p < 0.05$.

Figure S7. Inhibition of hepatocyte pyroptosis alleviates macrophage M1 polarization and metabolic reprogramming. **(A)** Heatmap of all genes involved in the top three up-regulated and down-regulated metabolism pathways according to Figure 9D. **(B)** iNOS fluorescence in primary macrophages showed that hepatocytes from CCl_4-injured mice significantly enhanced M1 polarization of macrophages compared to hepatocytes from control mice, while the absence of *Nlrp3* in hepatocytes reversed that (Scale bar: 25μm). **(C)** mRNA levels of *F4/80*, *Cd68* and *Nos2* in primary macrophages from above groups under co-culture with hepatocytes with different treatments (n = 3 per group). **(D, E)** mRNA and protein levels of GLUT1, HK1, HK2 and LDHA in primary macrophages from indicated group (n = 3 per group). **(F)** Glucose uptake, lactate production and ATP production in primary macrophages from indicated group (n = 5 per group). **(G)** Measurement of ECAR and OCR in above macrophages after co-culture with hepatocytes from mice with different treatments. All results are shown as mean ± SEM. $^{*}p < 0.05$, $^{\#}p < 0.05$.

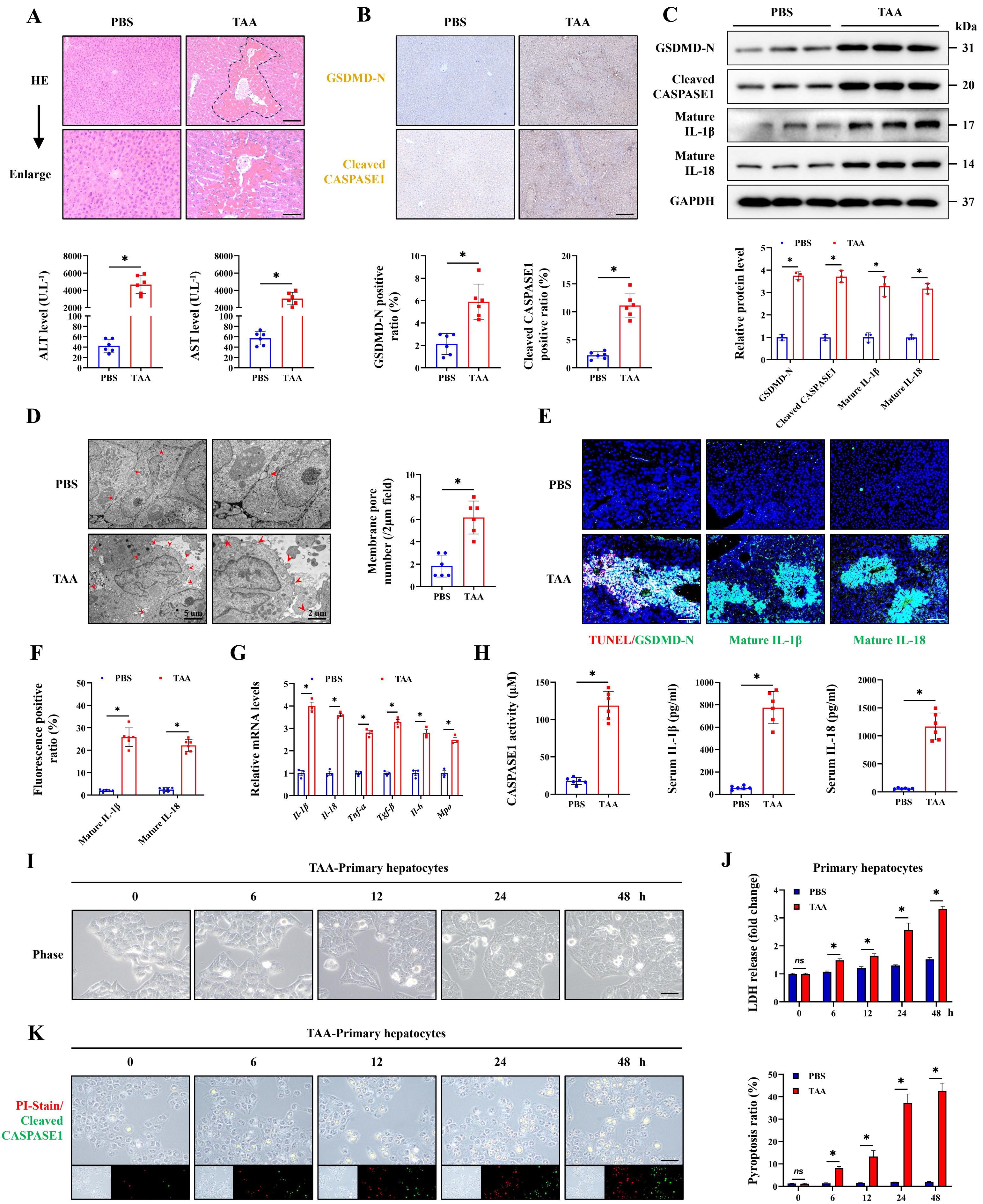

A
PBS
TAA
HE
Enlarge
ALT level (U.L-1)
AST level (U.L-1)
B
GSDMD-N
Cleaved CASPASE1
GSDMD-N positive ratio (%)
Cleaved CASPASE1 positive ratio (%)
C
kDa
GSDMD-N
Cleaved CASPASE1
Mature IL-1β
Mature IL-18
GAPDH
31
20
17
14
37
Relative protein level
D
Membrane pore number (/2μm field)
5 um
2 um
E
TUNEL/GSDMD-N
Mature IL-1β
Mature IL-18
F
Fluorescence positive ratio (%)
G
Relative mRNA levels
Il-1β
Il-18
Tnf-α
Tgf-β
Il-6
Mpo
H
CASPASE1 activity (μM)
Serum IL-1β (pg/ml)
Serum IL-18 (pg/ml)
I
TAA-Primary hepatocytes
0
6
12
24
48 h
Phase
J
Primary hepatocytes
LDH release (fold change)
ns
K
PI-Stain/ Cleaved CASPASE1
Pyroptosis ratio (%)

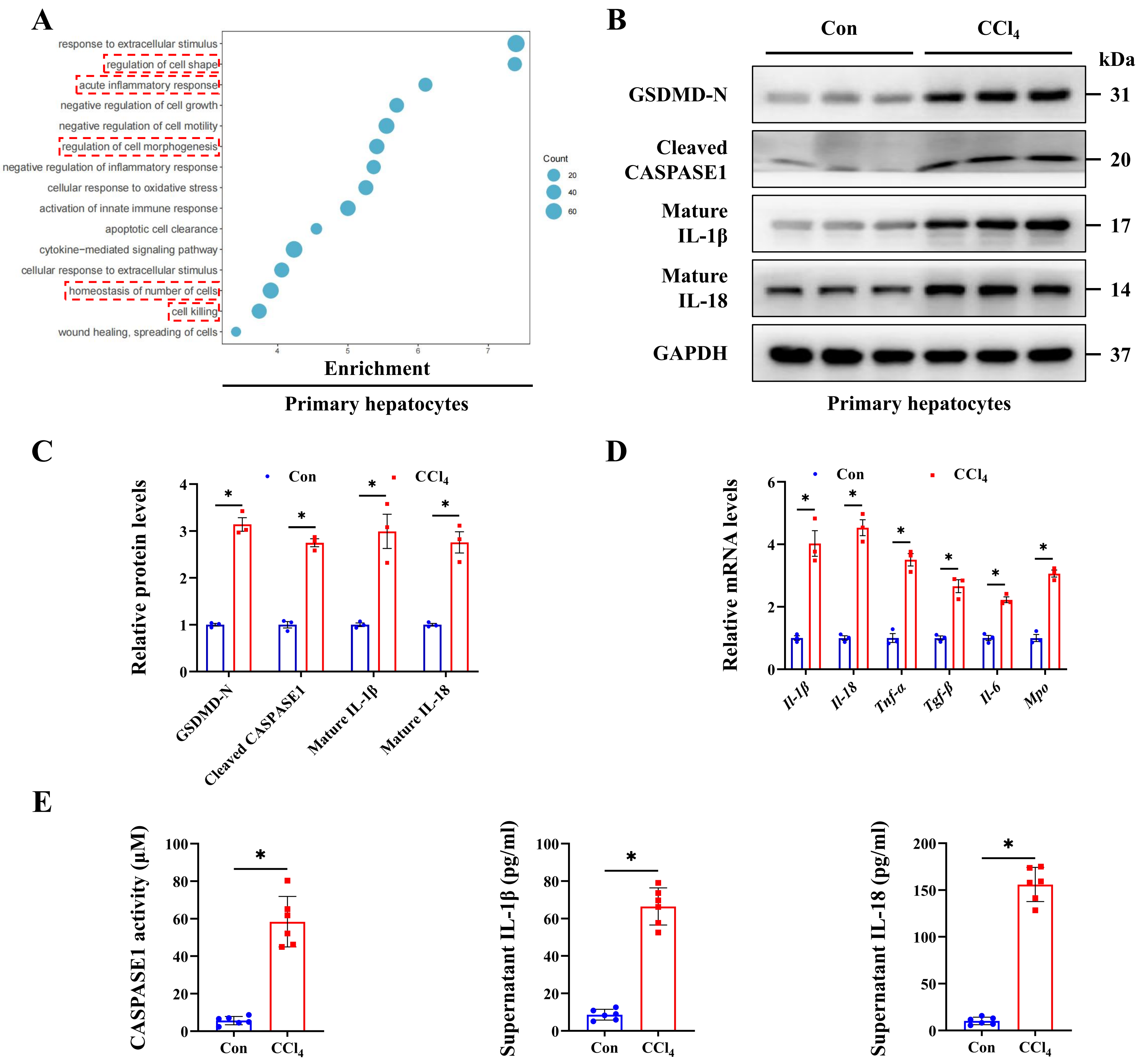

A
response to extracellular stimulus
regulation of cell shape
acute inflammatory response
negative regulation of cell growth
negative regulation of cell motility
regulation of cell morphogenesis
negative regulation of inflammatory response
cellular response to oxidative stress
activation of innate immune response
apoptotic cell clearance
cytokine−mediated signaling pathway
cellular response to extracellular stimulus
homeostasis of number of cells
cell killing
wound healing, spreading of cells
Count
20
40
60
4
5
6
7
Enrichment
Primary hepatocytes
B
Con
CCl4
kDa
GSDMD-N
31
Cleaved CASPASE1
20
Mature IL-1β
17
Mature IL-18
14
GAPDH
37
Primary hepatocytes
C
Con
CCl4
Relative protein levels
0
1
2
3
4
*
GSDMD-N
Cleaved CASPASE1
Mature IL-1β
Mature IL-18
D
Con
CCl4
Relative mRNA levels
0
2
4
6
*
Il-1β
Il-18
Tnf-α
Tgf-β
Il-6
Mpo
E
CASPASE1 activity (μM)
0
20
40
60
80
100
*
Con
CCl4
Supernatant IL-1β (pg/ml)
0
20
40
60
80
100
*
Con
CCl4
Supernatant IL-18 (pg/ml)
0
50
100
150
200
*
Con
CCl4

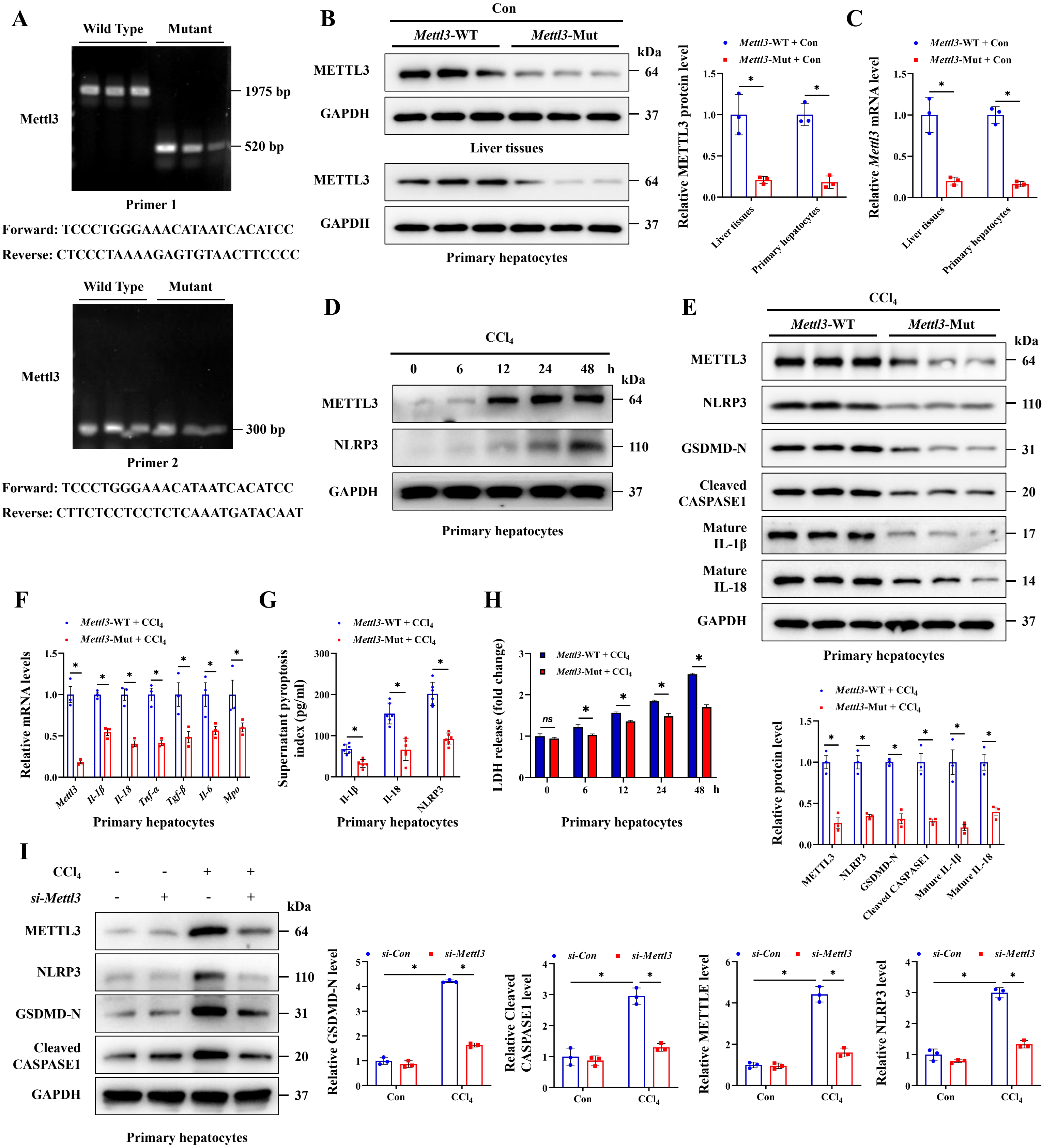
A
Wild Type
Mutant
Mettl3
1975 bp
520 bp
Primer 1
Forward: TCCCTGGGAAACATAATCACATCC
Reverse: CTCCCTAAAAGAGTGTAACTTCCCC
Wild Type
Mutant
Mettl3
300 bp
Primer 2
Forward: TCCCTGGGAAACATAATCACATCC
Reverse: CTTCTCCTCCTCTCAAATGATACAAT
B
Con
Mettl3-WT
Mettl3-Mut
kDa
METTL3
GAPDH
64
37
Liver tissues
METTL3
GAPDH
64
37
Primary hepatocytes
Relative METTL3 protein level
Mettl3-WT + Con
Mettl3-Mut + Con
Liver tissues
Primary hepatocytes
C
Relative Mettl3 mRNA level
Mettl3-WT + Con
Mettl3-Mut + Con
Liver tissues
Primary hepatocytes
D
CCl4
0 6 12 24 48 h
kDa
METTL3
NLRP3
GAPDH
64
110
37
Primary hepatocytes
E
CCl4
Mettl3-WT
Mettl3-Mut
kDa
METTL3
NLRP3
GSDMD-N
Cleaved CASPASE1
Mature IL-1β
Mature IL-18
GAPDH
64
110
31
20
17
14
37
Primary hepatocytes
Mettl3-WT + CCl4
Mettl3-Mut + CCl4
Relative protein level
METTL3
NLRP3
GSDMD-N
Cleaved CASPASE1
Mature IL-1β
Mature IL-18
F
Mettl3-WT + CCl4
Mettl3-Mut + CCl4
Relative mRNA levels
Mettl3
Il-1β
Il-18
Tnf-α
Tgf-β
Il-6
Mpo
Primary hepatocytes
G
Mettl3-WT + CCl4
Mettl3-Mut + CCl4
Supernatant pyroptosis index (pg/ml)
Il-1β
Il-18
NLRP3
Primary hepatocytes
H
Mettl3-WT + CCl4
Mettl3-Mut + CCl4
LDH release (fold change)
ns
0 6 12 24 48 h
Primary hepatocytes
I
CCl4
si-Mettl3
kDa
METTL3
NLRP3
GSDMD-N
Cleaved CASPASE1
GAPDH
64
110
31
20
37
Primary hepatocytes
si-Con
si-Mettl3
Relative GSDMD-N level
Relative Cleaved CASPASE1 level
Relative METTLE level
Relative NLRP3 level
Con
CCl4

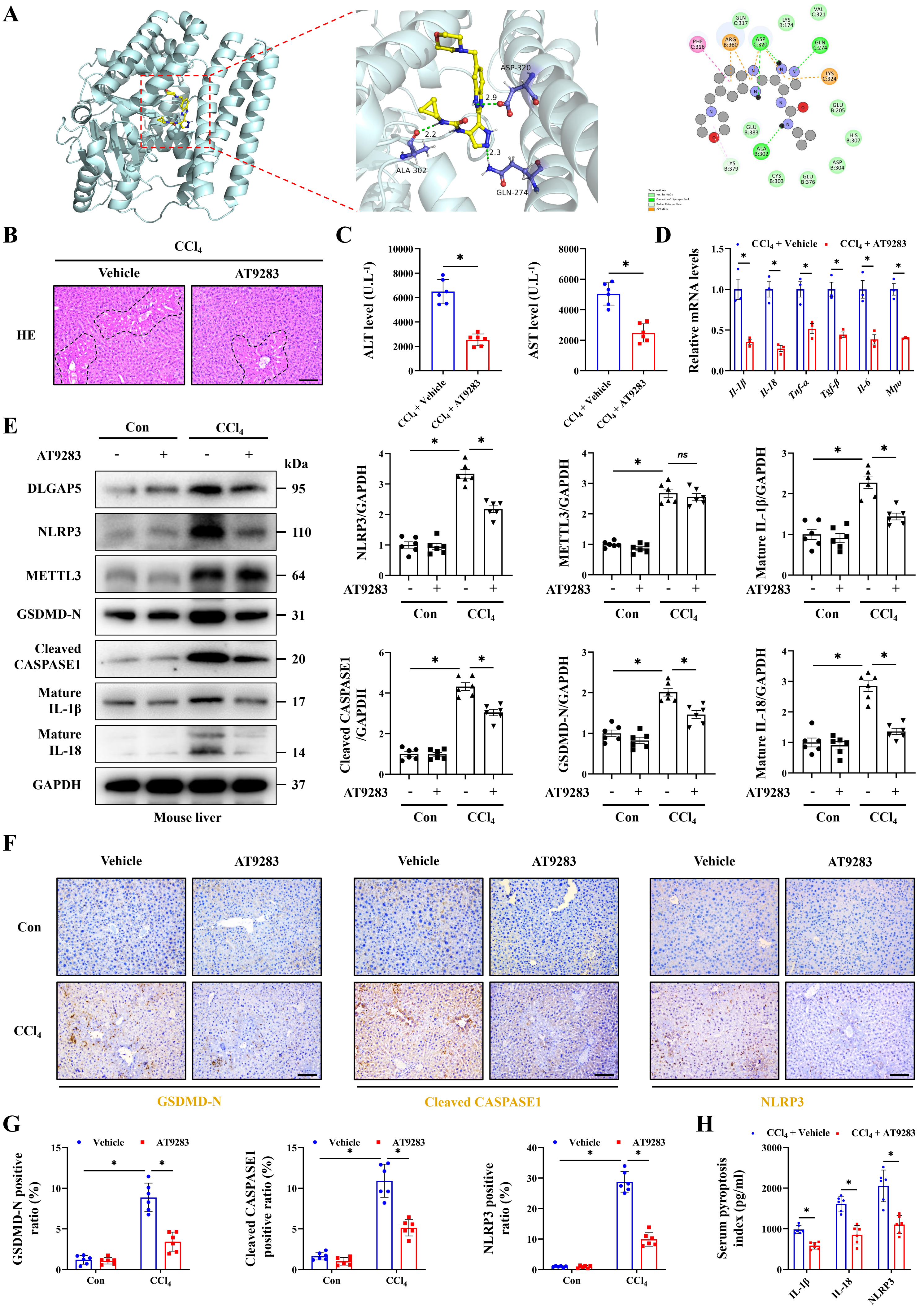

A
ASP-320
2.9
2.2
2.3
ALA-302
GLN-274
B
CCl4
Vehicle
AT9283
HE
C
ALT level (U.L-1)
AST level (U.L-1)
CCl4 + Vehicle
CCl4 + AT9283
D
Relative mRNA levels
Il-1β
Il-18
Tnf-α
Tgf-β
Il-6
Mpo
E
Con
CCl4
AT9283
kDa
DLGAP5
95
NLRP3
110
METTL3
64
GSDMD-N
31
Cleaved CASPASE1
20
Mature IL-1β
17
Mature IL-18
14
GAPDH
37
Mouse liver
NLRP3/GAPDH
METTL3/GAPDH
Mature IL-1β/GAPDH
Cleaved CASPASE1 /GAPDH
GSDMD-N/GAPDH
Mature IL-18/GAPDH
ns
F
Vehicle
AT9283
Con
CCl4
GSDMD-N
Cleaved CASPASE1
NLRP3
G
GSDMD-N positive ratio (%)
Cleaved CASPASE1 positive ratio (%)
NLRP3 positive ratio (%)
H
Serum pyroptosis index (pg/ml)
IL-1β
IL-18
NLRP3

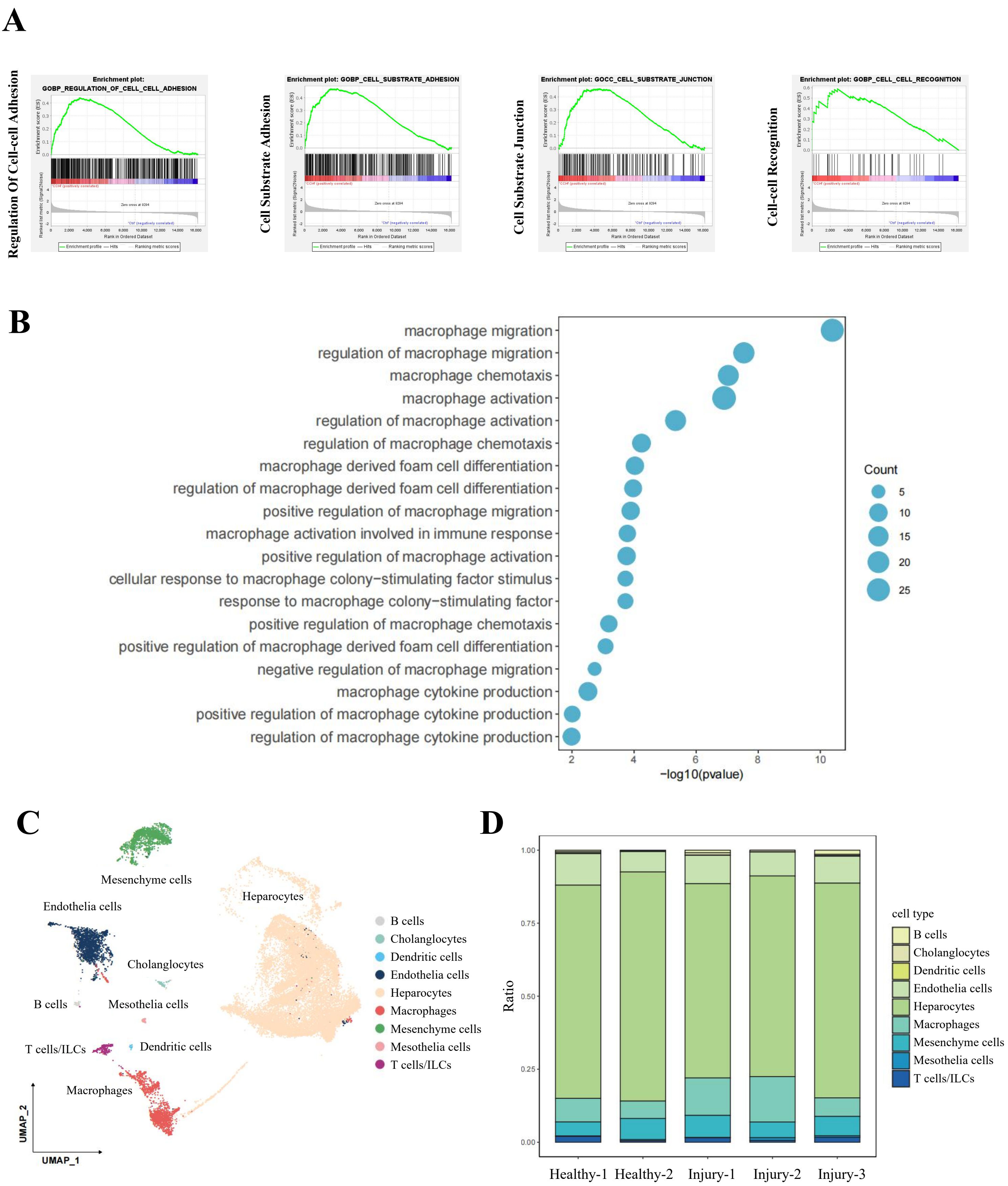

A
Regulation Of Cell-cell Adhesion
Enrichment plot: GOBP_REGULATION_OF_CELL_CELL_ADHESION
Cell Substrate Adhesion
Enrichment plot: GOBP_CELL_SUBSTRATE_ADHESION
Cell Substrate Junction
Enrichment plot: GOCC_CELL_SUBSTRATE_JUNCTION
Cell-cell Recognition
Enrichment plot: GOBP_CELL_CELL_RECOGNITION
Enrichment score (ES)
Ranked list metric (Signal2Noise)
Rank in Ordered Dataset
Enrichment profile
Hits
Ranking metric scores
B
macrophage migration
regulation of macrophage migration
macrophage chemotaxis
macrophage activation
regulation of macrophage activation
regulation of macrophage chemotaxis
macrophage derived foam cell differentiation
regulation of macrophage derived foam cell differentiation
positive regulation of macrophage migration
macrophage activation involved in immune response
positive regulation of macrophage activation
cellular response to macrophage colony−stimulating factor stimulus
response to macrophage colony−stimulating factor
positive regulation of macrophage chemotaxis
positive regulation of macrophage derived foam cell differentiation
negative regulation of macrophage migration
macrophage cytokine production
positive regulation of macrophage cytokine production
regulation of macrophage cytokine production
2
4
6
8
10
−log10(pvalue)
Count
5
10
15
20
25
C
Mesenchyme cells
Endothelia cells
Heparocytes
Cholanglocytes
B cells
Mesothelia cells
T cells/ILCs
Dendritic cells
Macrophages
UMAP_2
UMAP_1
B cells
Cholanglocytes
Dendritic cells
Endothelia cells
Heparocytes
Macrophages
Mesenchyme cells
Mesothelia cells
T cells/ILCs
D
Ratio
1.00
0.75
0.50
0.25
0.00
Healthy-1
Healthy-2
Injury-1
Injury-2
Injury-3
cell type
B cells
Cholanglocytes
Dendritic cells
Endothelia cells
Heparocytes
Macrophages
Mesenchyme cells
Mesothelia cells
T cells/ILCs

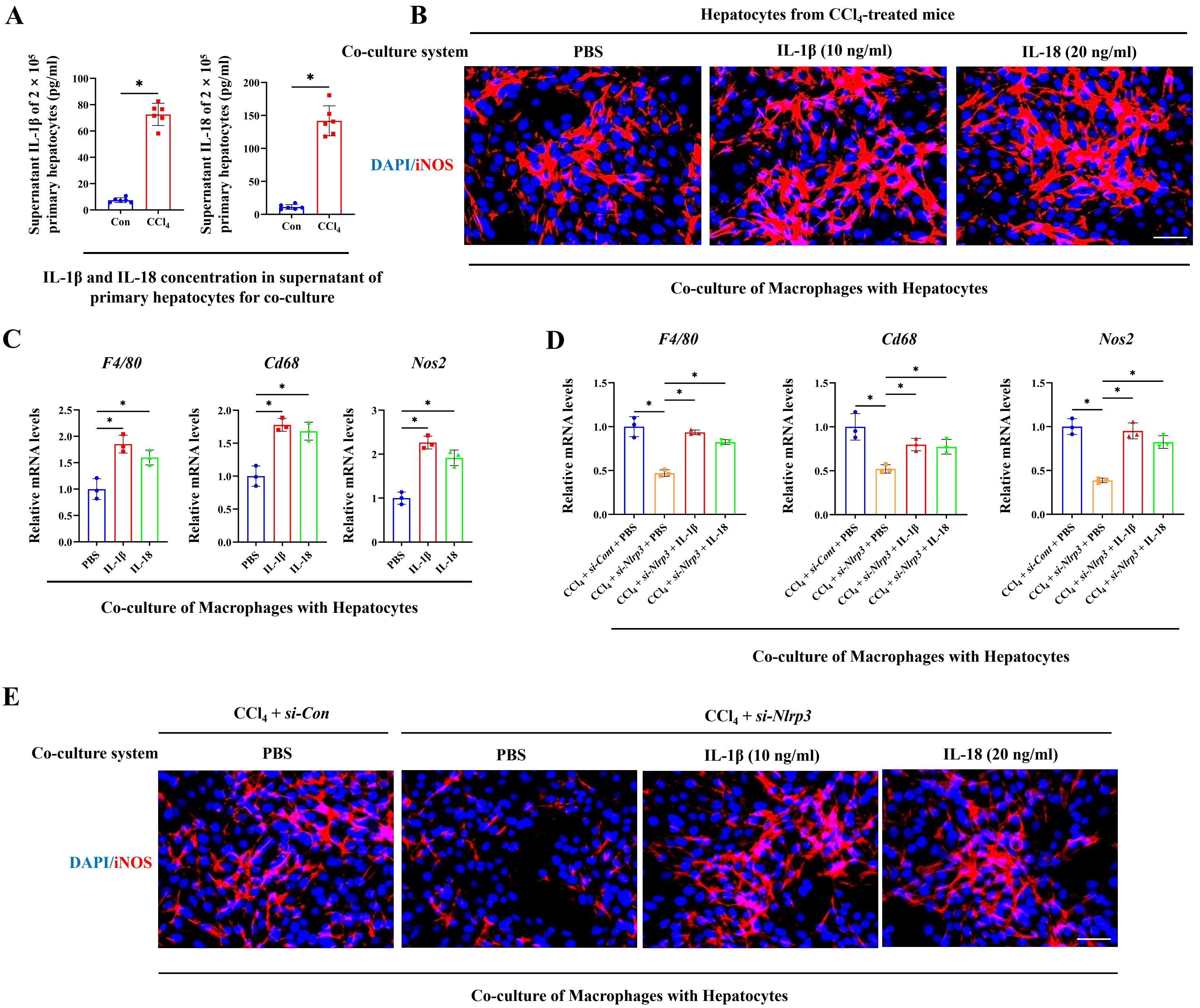
A
Supernatant IL-1β of 2 × 10^5 primary hepatocytes (pg/ml)
Con
CCl4
Supernatant IL-18 of 2 × 10^5 primary hepatocytes (pg/ml)
IL-1β and IL-18 concentration in supernatant of primary hepatocytes for co-culture
B
Hepatocytes from CCl4-treated mice
Co-culture system
PBS
IL-1β (10 ng/ml)
IL-18 (20 ng/ml)
DAPI/iNOS
Co-culture of Macrophages with Hepatocytes
C
F4/80
Cd68
Nos2
Relative mRNA levels
PBS
IL-1β
IL-18
Co-culture of Macrophages with Hepatocytes
D
F4/80
Cd68
Nos2
Relative mRNA levels
CCl4 + si-Cont + PBS
CCl4 + si-Nlrp3 + PBS
CCl4 + si-Nlrp3 + IL-1β
CCl4 + si-Nlrp3 + IL-18
Co-culture of Macrophages with Hepatocytes
E
CCl4 + si-Con
CCl4 + si-Nlrp3
Co-culture system
PBS
PBS
IL-1β (10 ng/ml)
IL-18 (20 ng/ml)
DAPI/iNOS
Co-culture of Macrophages with Hepatocytes

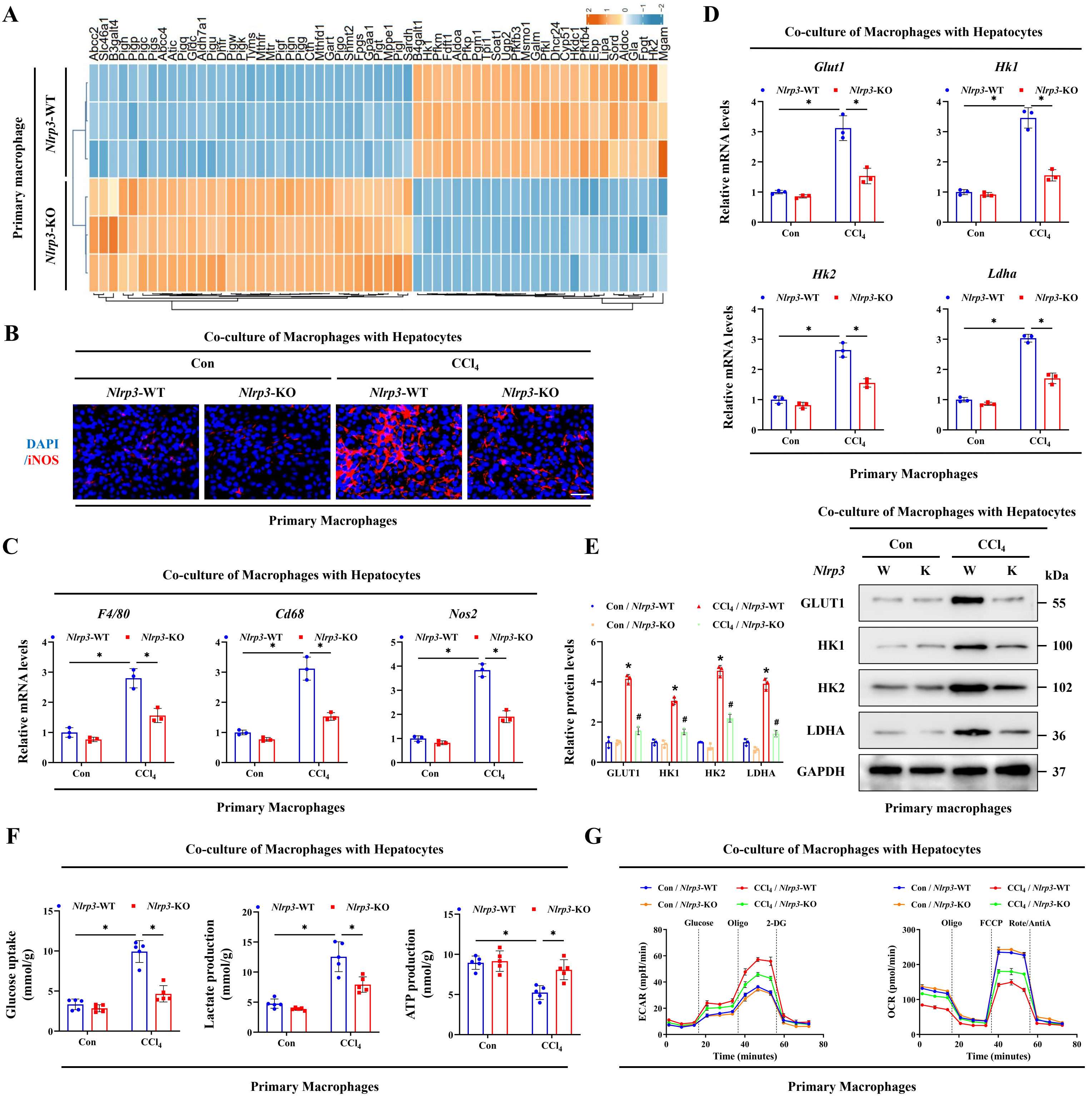
A
Abcc2 Slc46a1 B3galt4 Pigh Pigp Pigc Pigs Abcc4 Atic Pigq Gldc Aldh7a1 Pigu Dhfr Pigw Pigk Tyms Mthfr Mtr Pigf Pign Pigg Cth Mthfd1 Gart Pigo Shmt2 Fpgs Gpaa1 Pigt Mppe1 Pigl Sardh B4galt1 Hk1 Pfkm Fdft1 Aldoa Pfkp Pgm1 Tpi1 Soat1 Ugp2 Pfkfb3 Msmo1 Galm Pfkl Dhcr24 Cyp51 Hkdc1 Pfkfb4 Ebp Lipa Sord Aldoc Gla Fpgt Hk2 Mgam
Primary macrophage
Nlrp3-WT
Nlrp3-KO
B
Co-culture of Macrophages with Hepatocytes
Con
CCl4
Nlrp3-WT
Nlrp3-KO
DAPI
/iNOS
Primary Macrophages
C
Co-culture of Macrophages with Hepatocytes
F4/80
Cd68
Nos2
Relative mRNA levels
Nlrp3-WT
Nlrp3-KO
Con
CCl4
Primary Macrophages
D
Co-culture of Macrophages with Hepatocytes
Glut1
Hk1
Hk2
Ldha
Relative mRNA levels
Nlrp3-WT
Nlrp3-KO
Con
CCl4
Primary Macrophages
E
Co-culture of Macrophages with Hepatocytes
Con / Nlrp3-WT
CCl4 / Nlrp3-WT
Con / Nlrp3-KO
CCl4 / Nlrp3-KO
Relative protein levels
GLUT1
HK1
HK2
LDHA
Con
CCl4
Nlrp3
W
K
kDa
GLUT1 55
HK1 100
HK2 102
LDHA 36
GAPDH 37
Primary macrophages
F
Co-culture of Macrophages with Hepatocytes
Nlrp3-WT
Nlrp3-KO
Glucose uptake (mmol/g)
Lactate production (mmol/g)
ATP production (nmol/g)
Con
CCl4
Primary Macrophages
G
Co-culture of Macrophages with Hepatocytes
Con / Nlrp3-WT
CCl4 / Nlrp3-WT
Con / Nlrp3-KO
CCl4 / Nlrp3-KO
Glucose
Oligo
2-DG
ECAR (mpH/min)
Time (minutes)
Oligo
FCCP
Rote/AntiA
OCR (pmol/min)
Time (minutes)
Primary Macrophages
